# The effect of continuous long-term illumination with visible light in different spectral ranges on mammalian cells

**DOI:** 10.1038/s41598-024-60014-9

**Published:** 2024-04-24

**Authors:** Finn Dani, Kathleen Schütz, Ezgi Dikici, Anne Bernhardt, Anja Lode

**Affiliations:** Centre for Translational Bone, Joint and Soft Tissue Research, Faculty of Medicine, Technical University Dresden, Dresden, Germany

**Keywords:** Cell biology, Cellular imaging, Biomedical engineering

## Abstract

One of the biggest challenges in tissue engineering and regenerative medicine is to ensure oxygen supply of cells in the (temporary) absence of vasculature. With the vision to exploit photosynthetic oxygen production by microalgae, co-cultivated in close vicinity to oxygen-consuming mammalian cells, we are searching for culture conditions that are compatible for both sides. Herein, we investigated the impact of long-term illumination on mammalian cells which is essential to enable photosynthesis by microalgae: four different cell types—primary human fibroblasts, dental pulp stem cells, and osteoblasts as well as the murine beta-cell line INS-1—were continuously exposed to warm white light, red or blue light over seven days. We observed that illumination with red light has no adverse effects on viability, metabolic activity and growth of the cells whereas exposure to white light has deleterious effects that can be attributed to its blue light portion. Quantification of intracellular glutathione did not reveal a clear correlation of this effect with an enhanced production of reactive oxygen species. Finally, our data indicate that the cytotoxic effect of short-wavelength light is predominantly a direct effect of cell illumination; photo-induced changes in the cell culture media play only a minor role.

## Introduction

Light is a form of electromagnetic irradiation whose energy causes responses in natural biological systems. Prominent examples are the photosynthesis in green plants, algae and some specific bacteria, in which the light energy is converted into chemical energy stored in organic compounds, or the synthesis of vitamin D from its precursor in the human skin by sun light exposure. In addition, organisms have developed protective mechanisms to prevent damage caused by high energy input from extensive exposure to sun light such as pigment formation/darkening in plants (colour pigments like anthocyanin, xanthophylls) or human skin (melanin).

Most mammalian tissues and organs—except for skin and eyes—are not exposed to light in their physiological environment and therefore, not adapted to it. However, in fundamental research or in artificial biological systems, mammalian cells can come in contact with light. In live cell fluorescence microscopy, for example, cells are exposed to excitation light of a certain wavelength. In optogenetic approaches, light of a certain wavelength is used as an optical actuator to switch on or off specific activities in the genetically modified cells, thereby modulating their functionality^1,2^. However, in both technologies the light has been described to affect cell physiology—for instance, excessive intensities of blue light caused altered gene expression, reduced cell motility, diminished cell viability and mitosis as well as increased apoptosis^3–8^. To avoid or at least minimize such side effects, called phototoxicity, it is of importance to assess the effect of light on naturally non light-adapted cells with the aim to establish harmless illumination regimes. There is evidence that illumination at lower intensities or longer wavelengths carries a considerably lower risk of phototoxic cell damage or impairment^4,5,7,9^.

Against the background of a therapeutic application in medicine and dentistry, to stimulate tissue repair processes and to reduce pain and inflammation, photobiomodulation is another research field in which cells are exposed to light^10^. For many cell types including fibroblasts, osteoblasts and mesenchymal stem cells, the cyclic short-term exposure—in the range of fractions of a second up to a few minutes/hours over several days—to red/infrared light of low intensity (laser or LED) has been shown to increase the viability and have a stimulatory effect on cellular activities such as proliferation, differentiation, and secretion of signalling factors^11–15^. A few studies also demonstrated a photobiomodulatory effect of blue or green light on cell metabolism, but only at low energy densities^16,17^.

In the research field of Tissue Engineering and Regenerative Medicine, one of the biggest challenges is to ensure a sufficient oxygen supply of cells in the (temporary) absence of vasculature, e. g. after transplantation or within in vitro cultured 3D constructs. An exciting concept is to exploit photosynthetic oxygen production by microalgae or cyanobacteria for delivery to the oxygen-consuming cells^18–21^. Therefore, cocultures of mammalian cells with photosynthetically active cells are established and investigated; first studies demonstrated both, their great potential and their limitations ^22–26^. A crucial aspect of the development of a cultivation regime compatible with both cell types is the illumination, which is essential to enable photosynthesis. In contrast to the above-mentioned applications, which use light at a very specific wavelength and relatively short incubation times, such a coculture approach rather requires a long-term, continuous light exposure to ensure a continuous oxygen supply. In addition, photosynthetically active microorganisms are adapted to sun light with its broad spectrum and therefore, warm white light is the first choice for this approach ^18,19,22,23,25,26^.

Our group is working on the establishment of bio-printed coculture constructs in which insulin-producing beta-cells are cultivated in close proximity to oxygen-producing microalgae. Preliminary investigations indicated a negative effect of continuous white light illumination on the murine beta-cell line INS-1 ^27^. Therefore, the aim of the present study was to investigate the effect of continuous, long-term exposure to warm white light (full spectrum visible light) in direct comparison to red and blue light on the viability, morphology and proliferation of different mammalian cell types. Based on our previous observation and above-mentioned findings described in literature, we hypothesized that long-term illumination with white light is less tolerable for mammalian cells than red light of the same intensity because of its blue light portion. In addition to the INS-1 beta-cell line, we included with human dermal fibroblasts, human osteoblasts, and human mesenchymal stem cells (isolated from dental pulp) primary cell types which have been frequently studied in the course of photobiomodulatory approaches. Besides testing our hypothesis, we investigated the question whether there is a correlation between (reduced) viability and reactive oxygen species (ROS) production under light exposure, as the involvement of ROS in cytotoxic responses to blue light has been suggested^3,28^. Finally, we examined whether light exposure of the cell culture media leads to the generation of cytotoxic products affecting the cells.

## Material and methods

### Cell culture

Normal human dermal fibroblasts (NHDF), purchased from Promocell (Heidelberg, Germany), were cultivated in Dulbecco’s modified Eagle’s medium (DMEM; Gibco, Life Technologies, Germany) containing 10% fetal calf serum (FCS; Corning, NY, USA), 100 U/mL penicillin, and 100 μg/mL streptomycin (P/S; Gibco, Life Technologies). Human dental pulp stem cells (DPSC), isolated from deciduous teeth as previously published^29^, were provided by the Department of Maxillofacial Surgery at University Hospital Carl Gustav Carus Dresden. DPSC of two different donor origins were cultivated in DMEM containing 20% FCS and P/S. Human osteoblasts (hOB) were isolated from femoral heads of two osteoarthritic patients undergoing total hip replacement at the University Hospital Carl Gustav Carus Dresden (Germany)^30^. The hOB were expanded in alpha-MEM (Minimal Essential Medium, alpha-Modification; Gibco, Life Technologies) containing 10% FCS and P/S (100 U/mL penicillin, 100 μg/mL streptomycin). All primary cells were used in passage 5 for the experiments. The rat insulinoma derived beta-cell line INS-1, a kind donation from Prof. emeritus Claes B. Wollheim (MD Department of Cell Physiology and Metabolism, University Medical Center 1, Geneva, Switzerland)^31^. was cultivated in RPMI-1640 medium containing 11.1 mmol/L D glucose (Gibco, Life Technologies) and supplemented with 10% FCS, P/S (100 U/mL penicillin, 100 μg/mL streptomycin), 10 mmol/L HEPES (Carl Roth, Germany), 2 mmol/L L-glutamine (Merck Millipore, Biochrom, Germany), 1 mmol/L sodium pyruvate (AppliChem, Germany), and 50 µmol/L 2-mercaptoethanol (Sigma-Aldrich, Germany).

The ethics commission of the TU Dresden approved the application of DPSC and hOB (DPSC: EK 106,042,010 and hOB: EK 303,082,014), all experiments were performed in accordance with relevant guidelines and regulations. Informed consent was obtained from all participants and/or their legal guardians.

### Light sources

**Figure 1:**
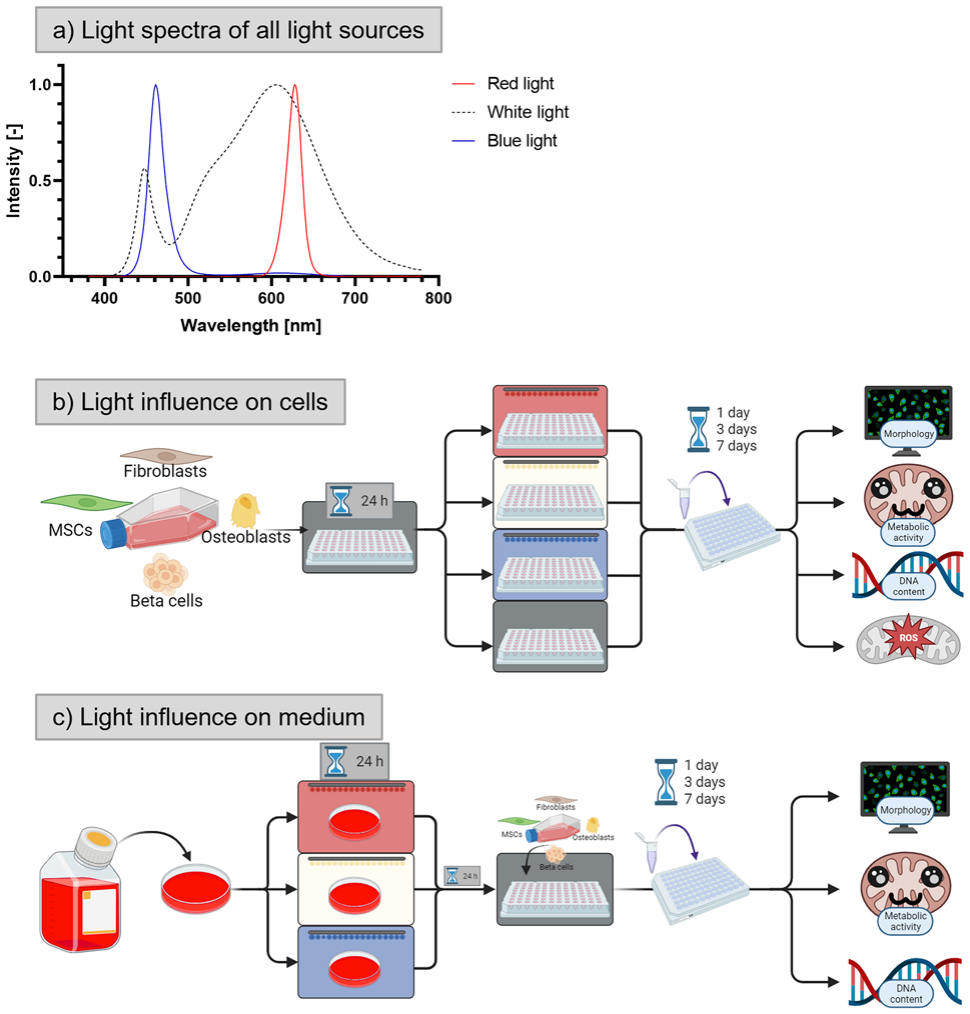
Light spectra of the used light sources (**a**) and schematic description of the analysis of light influence on cells (**b**) and on cell culture medium (**c**).

Cells exposed to light were cultivated in a Multitron incubator with CO_2_ supply (Infors HT, Switzerland). For white light, the built-in LED lights were used for a homogenous illumination from above, resulting in a light intensity of 80 µmol m^−2^ s^−1^ (2 mW/cm^2^) at the height of the cell layer. This intensity was chosen to be as low as possible to minimize the impact on mammalian cells while still allow photosynthetic oxygen production. Red and blue light were provided by an LED strip (LLV-shop, Germany), also providing homogenous illumination from above in the same intensity (measured in µmol m^−1^ s^−1^). Samples cultivated in darkness were kept in a HERAcell CO_2_ incubator (Thermo Fisher Scientific). Standard cell culture conditions (37 °C, 5% CO_2_) were applied in both incubators. Light spectra were measured using an LI-180 spectrometer (LI-COR Biosciences GmbH, Germany) and are shown in Fig. 1a.

### Experimental setup

For each cell type, cultivation took place under three different conditions: (1) In the ‘control’ group, the cells were cultivated with their respective medium in the dark; (2) in the ‘light’ group, the cells were cultivated with their respective medium under continuous light exposure (white, red, or blue light); and (3) in the ‘light-treated medium’ group, the cells were cultivated in the dark with their respective medium which was illuminated with red, white, or blue light for 24 h directly prior to adding it to the cells. A fourth group served as positive control in the experiments for ROS analysis, to validate the functionality of the MCB assay (see 2.5.): The cells were cultivated with their respective medium, which was supplemented with 100 µM (INS-1) or 10 µM (all other cell types) CuCl_2_, in the dark. Every experiment was performed having a control group cultivated in darkness and using white light and one other light source to ensure comparability between the experiments and to minimise the impact of other influencing factors (i.e. day-specific cell behaviour, differences in temperature and light control). Cells were seeded into 96-well plates (3.2 × 10^3^ cells/well for NHDF and DPSC, 10 × 10^3^ cells/well for hOB and 41 × 10^3^ cells/well for INS-1; 100 µL cell culture medium per well) and allowed to attach overnight in the dark. On the next day (day 0), the medium was changed, and the plates were brought into their respective experimental environment. After three days, the medium was changed again for samples cultivated until day 7.

### Analysis of metabolic activity

The WST-1 cell proliferation kit (ab65473, Abcam, United Kingdom) is based on the cleaving of the tetrazolium salt WST-1 to a soluble formazan by mitochondrial dehydrogenases. On day 1, 3, and 7 of culture, 10 µL of WST-1 working solution were added to each well; after 60 min of incubation, absorbance was measured at 440 nm (Infinite M200 Pro, Tecan, Switzerland). Thereafter, the cells were washed once with phosphate buffered saline (PBS) before adding fresh medium and further cultivation. With this method, the same cells were analysed on each time point of the experiment to exclude variation between samples as a source of error. All results were normalised to the first day values of the control group cultured in darkness.

### Measurement of glutathione levels

Reduced levels of intracellular glutathione (GSH) can be interpreted as an indirect measurement of ROS concentration. GSH levels were measured using the optimised monochlorbimane (MCB) assay as previously described^32,33^. On day 1, 3, and 7 of culture, 240 µM MCB (Sigma-Aldrich, Germany) in PBS were added directly to the medium to achieve a final concentration of 40 µM and fluorescence was recorded immediately at Ex/Em 394/490 nm over 12 min using a multiplate reader (Tecan). All results were normalised to those of the control group cultured in darkness on day 1. Furthermore, GSH levels were related to the DNA content of the respective sample.

### DNA analysis

After GSH measurement, the DNA content of the cells was quantified using the blue fluorescing nucleic acid dye Hoechst 33,258 (FluoReporter™ Blue Fluorometric dsDNA Quantitation Kit (F2962), Invitrogen™, Thermo Fisher Scientific, MA, USA). For cell lysis, the medium was replaced by 100 µL dH_2_O before the samples were frozen at − 20 °C. Later, they were incubated for one hour at 37 °C before 100 µL of the staining solution (prepared as described by the manufacturer) were added immediately before measuring the fluorescence at Ex/Em 360/460 nm using a multiplate reader (Tecan). All results were normalised to the first day values of the control group cultured in darkness.

### Morphological analysis

On day 1, 3, and 7 of culture, four samples of each group were washed once with PBS before being fixed using 4% formaldehyde in PBS for 30 min. After permeabilisation (0.1% Triton-X in PBS for five minutes), the samples were incubated in 1% bovine serum albumin (BSA) in PBS overnight followed by staining with 1 µL/mL DAPI (Gibco Life Technologies, stock solution prepared to the manufacturer’s protocol) and 25 µL/mL Phalloidin iFluor 488 (Invitrogen, USA, stock solution prepared to the manufacturer’s protocol) in 1% BSA/PBS solution for 60 min. The cells were imaged at 10 × magnification using a Keyence AIO Fluorescent Microscope BZ-X800 (Keyence, Japan).

### Statistical analysis

All results were evaluated by one-way Analysis of Variance (ANOVA), followed by Dunnetts multiple comparison test with GraphPad Prism 9 software. At every time point, the comparison was done between dark control and the experimental groups. Significant differences were assumed at *p < 0.05; **p < 0.01; ***p < 0.001; ****p < 0.0001.

## Results

Figure 1 provides an overview of the experiments conducted in this study. First, the influence of continuous illumination during cell culture was comparatively investigated for white, red, and blue light (Fig. 1a) of equal intensity in contrast to the dark control. After seeding the cells into cell culture plates, they were allowed to attach in the dark. 24 h later, the cells were brought into their respective experimental environment (red light, white light, blue light, dark) and cultured for seven days. On day 1, 3, and 7 of culture, cell morphology and density were evaluated by fluorescence microscopy after staining of the actin cytoskeletons (green) and cell nuclei (blue). Proliferation of the cells was assessed by means of cell number development over time, quantified by measuring the DNA content. The viability of the cells was examined by assessing their metabolic activity using the WST-1 assay. Formation of ROS was analysed via determination of the GSH level using the MCB assay (Fig. 1b).

The influence of light-exposed cell culture medium on the cells was investigated by cultivating them in the dark with medium which was illuminated with red, white, or blue light for 24 h prior to adding it to the cells; medium without light exposure served as dark control. On day 1, 3, and 7 of culture, cell morphology, proliferation and viability were analyzed by fluorescence microscopy and measurement of DNA content as well as metabolic activity (Fig. 1c).

All experiments were carried out with human fibroblasts (NHDF), human mesenchymal stem cells from dental pulp (DPSC obtained from two donors), human osteoblasts (hOB obtained from two donors) and the murine beta-cell line INS-1. For a better illustration of the information presented, all quantitative values were normalised to those of the dark control group measured on day 1.

### Influence of long-term red and white light illumination

#### Normal human dermal fibroblasts

**Figure 2:**
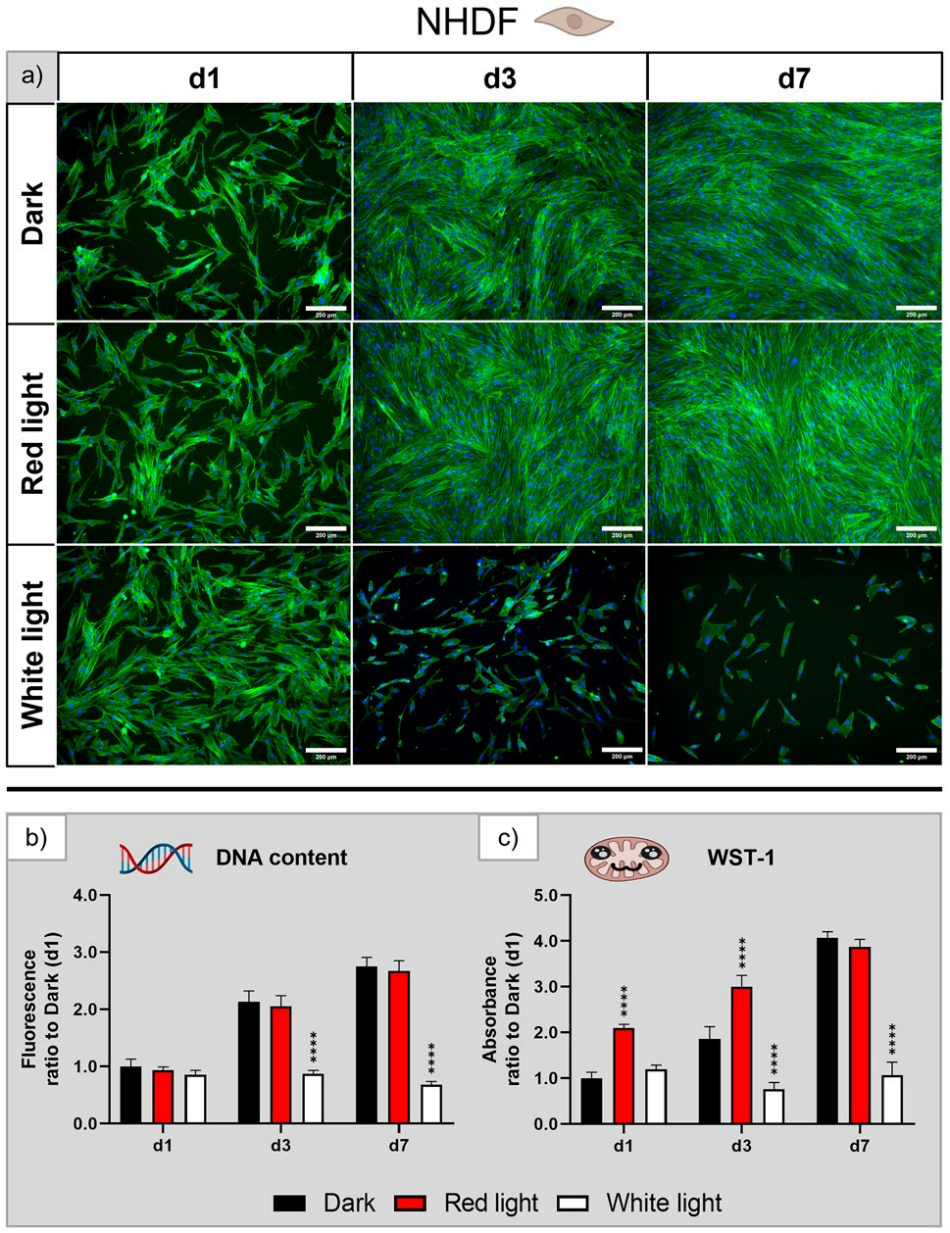
Influence of red and white light illumination on NHDF during cultivation over seven days: (**a**) microscopy images of fluorescence staining (green—actin cytoskeletons, blue—cell nuclei), scale bar: 200 µm; (**b**) DNA content and (**c**) metabolic activity, both normalised to the dark control on day 1. n = 4, mean ± SD; p-values: ****p < 0.0001.

Fluorescence microscopic images of NHDF (Fig. 2a) indicated proliferation of the cells in the dark control and under red light illumination. However, after exposure to white light for three and seven days the number of attached cells decreased dramatically. This effect is also reflected in the DNA content (Fig. 2b). DNA content increased twofold from day 1 to day 3 and threefold to day 7 for both dark control and red light without statistically significant differences between both groups. In contrast, cell number under white light illumination did not increase, leading to a significantly lower cell number after three and seven days of cultivation compared to the other two groups. The cells showed the typical elongated morphology in the dark control and under red light illumination, whereas under white light illumination the cells appeared less elongated. The WST-1 assay (Fig. 2c) indicated significantly reduced metabolic activity in cells cultured under white light for three and seven days. Also of note is the significantly increased activity of cells exposed to red light after one and three days of cultivation compared to the dark control, suggesting a possible beneficial effect of red light on human fibroblasts. By the end of the experiment, the metabolic activity of the dark and the red light groups has equalised, both reaching a fourfold increase in activity, corresponding with the similar increase in cell numbers.

In summary, the three complementary methods indicated that exposure to red light has no or even a positive effect, while white light illumination has a significant negative impact on morphology, activity, and growth of NHDF. These observations were confirmed by two repeating experiments (Supplementary Fig. S1).

#### Human dental pulp stem cells

**Figure 3:**
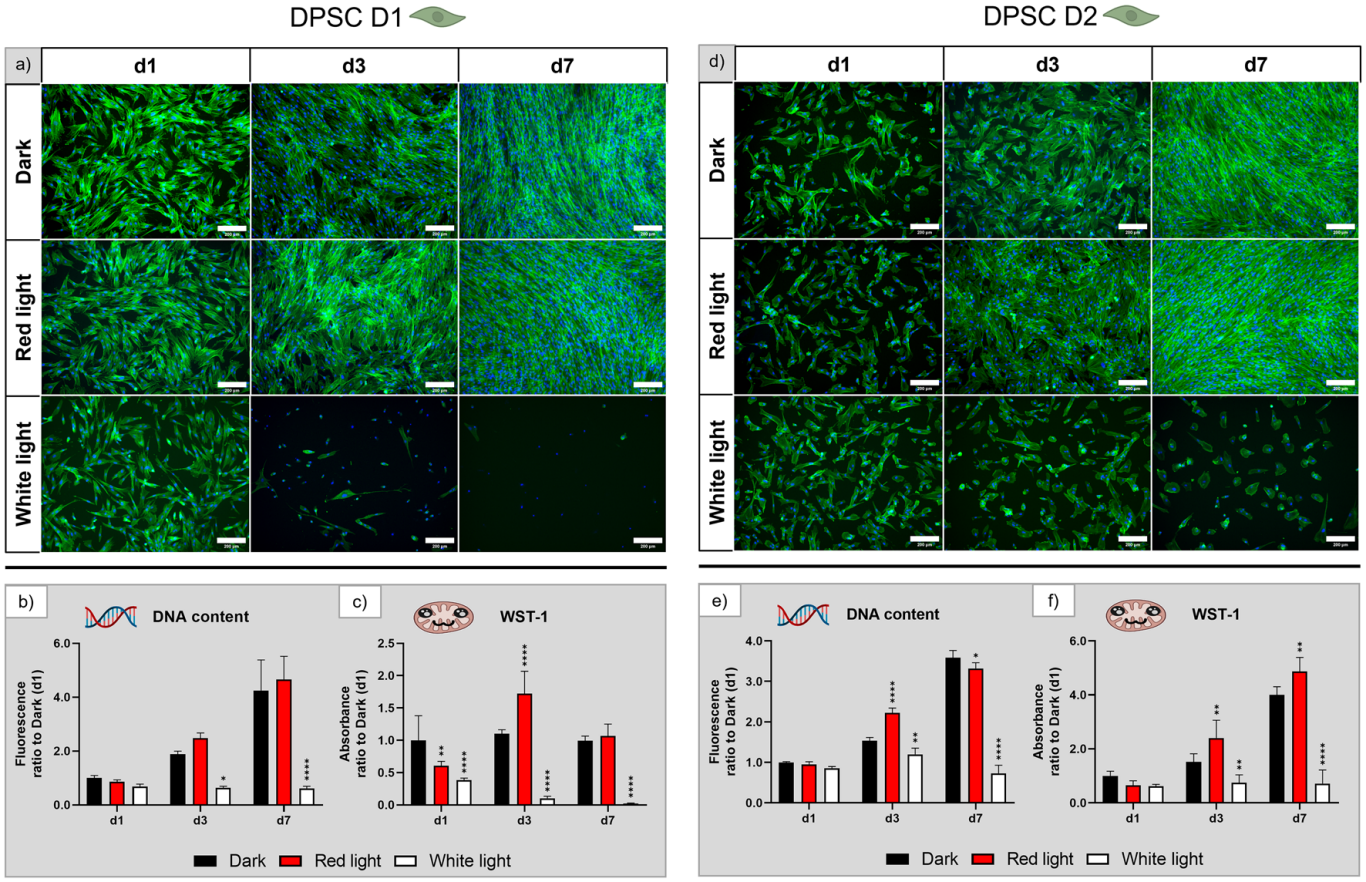
Influence of red and white light illumination on DPSC derived from donor 1 (D1) and donor 2 (D2) during cultivation over seven days: (**a**) and (**d**) microscopy images of fluorescence staining (green—actin cytoskeletons, blue—cell nuclei), scale bar: 200 µm; (**b**) and (**e**) DNA content and (**c**) and (**f**) metabolic activity, all normalised to the dark control on day 1. n = 4, mean ± SD; p-values: *p < 0.05; **p < 0.01; ****p < 0.0001.

Fluorescence microscopic images of human DPSC, derived from two different donors (Fig. 3a,d), demonstrate proliferation of DPSC under red light illumination similar to the dark control. Under white light illumination, the number of attached cells was drastically reduced by day 3 and even more by day 7. The elongated morphology of the cells was identical in cultures grown in darkness and under red light illumination, whereas in white light only a few elongated cells were visible on day 3 and even less on day 7. The DNA content (Fig. 3b,e) was on a constantly low level for cells cultured in white light, whereas it increased up to fourfold in the dark control and red light groups until day 7. The WST-1 assay (Fig. 3c,f) indicated drastically reduced metabolic activity for DPSC exposed to white light: For cells of donor 1 (D1), it was significantly decreased already after one day (Fig. 3c) while for cells of donor 2 (D2), a significant decrease was detected after three and seven days of culture (f). At some time points (day 3 for D1, day 3 and 7 for D2), metabolic activity of DPSC was significantly higher under red light illumination compared to the dark control.

In summary, red light illumination has no negative or even a positive effect on DPSC, white light illumination has a strong negative impact on morphology, activity, and growth of DPSC. In two repeating experiments, the different influence of red and white light illumination on the DPSC was confirmed, however, the differences in the metabolic activity were less pronounced (Supplementary Fig. S2 and S3).

#### Human osteoblasts

**Figure 4:**
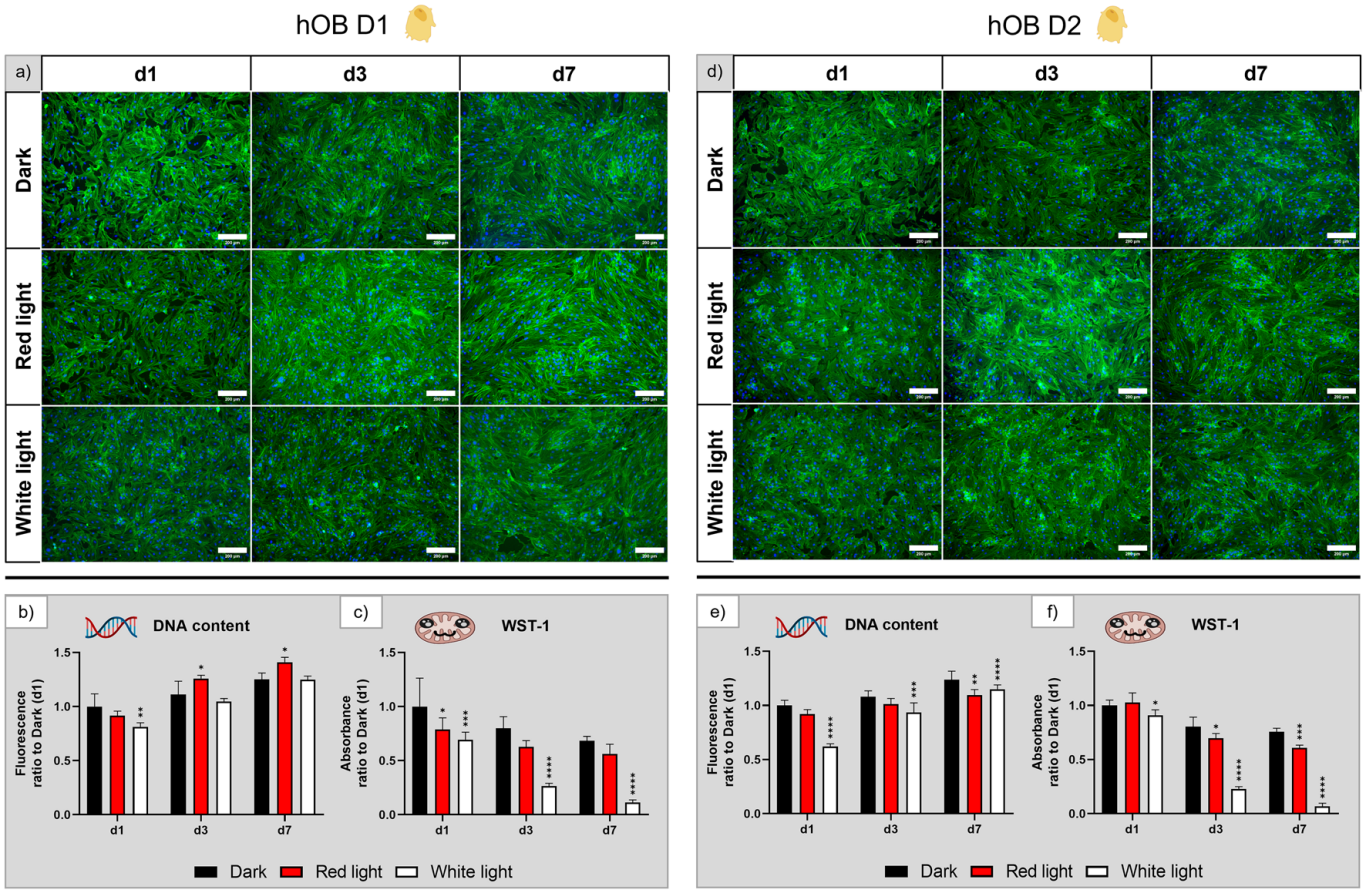
Influence of red and white light illumination on hOB derived from donor 1 (D1) and donor 2 (D2) during cultivation over seven days: (**a**) and (**d**) microscopy images of fluorescence staining (green—actin cytoskeletons, blue—cell nuclei), scale bar: 200 µm; (**b**) and (**e**) DNA content and (**c**) and (**f**) metabolic activity, all normalised to the dark control on day 1. n = 4, mean ± SD; p-values: *p < 0.05; **p < 0.01; ***p < 0.001; ****p < 0.0001.

Fluorescence microscopic images of hOB, derived from two different donors (Fig. 4a,d), demonstrate attached and spread cells at all examined time points and there were no differences in area coverage and morphology visible between the three experimental groups. The DNA content (Fig. 4b,e) is consistent with these findings. While the overall proliferation was not high (only a 1.25-fold increase in DNA content for the dark control group), both red and white light exposed groups showed the same trend of increasing cell numbers. For cells of donor 1 (D1), red light exposed samples showed a significantly higher DNA content on day 3 and day 7 compared to the dark control, while for cells of donor 2 (D2), the DNA content was significantly reduced under white light compared to the dark control. The WST-1 assay (Fig. 4c,f) revealed a decrease of the metabolic activity for white light-exposed cells, which had a significantly lower metabolic activity than the other two groups throughout the experiment.

In summary, morphology and proliferation of hOB is not affected by red or white light illumination. However, cells of both donors had a strongly reduced metabolic activity under the influence of white light illumination that was not detectable in the red light group—this was confirmed by two repeating experiments (Supplementary Figs. S4 and S5).

#### Murine cell line INS-1

**Figure 5:**
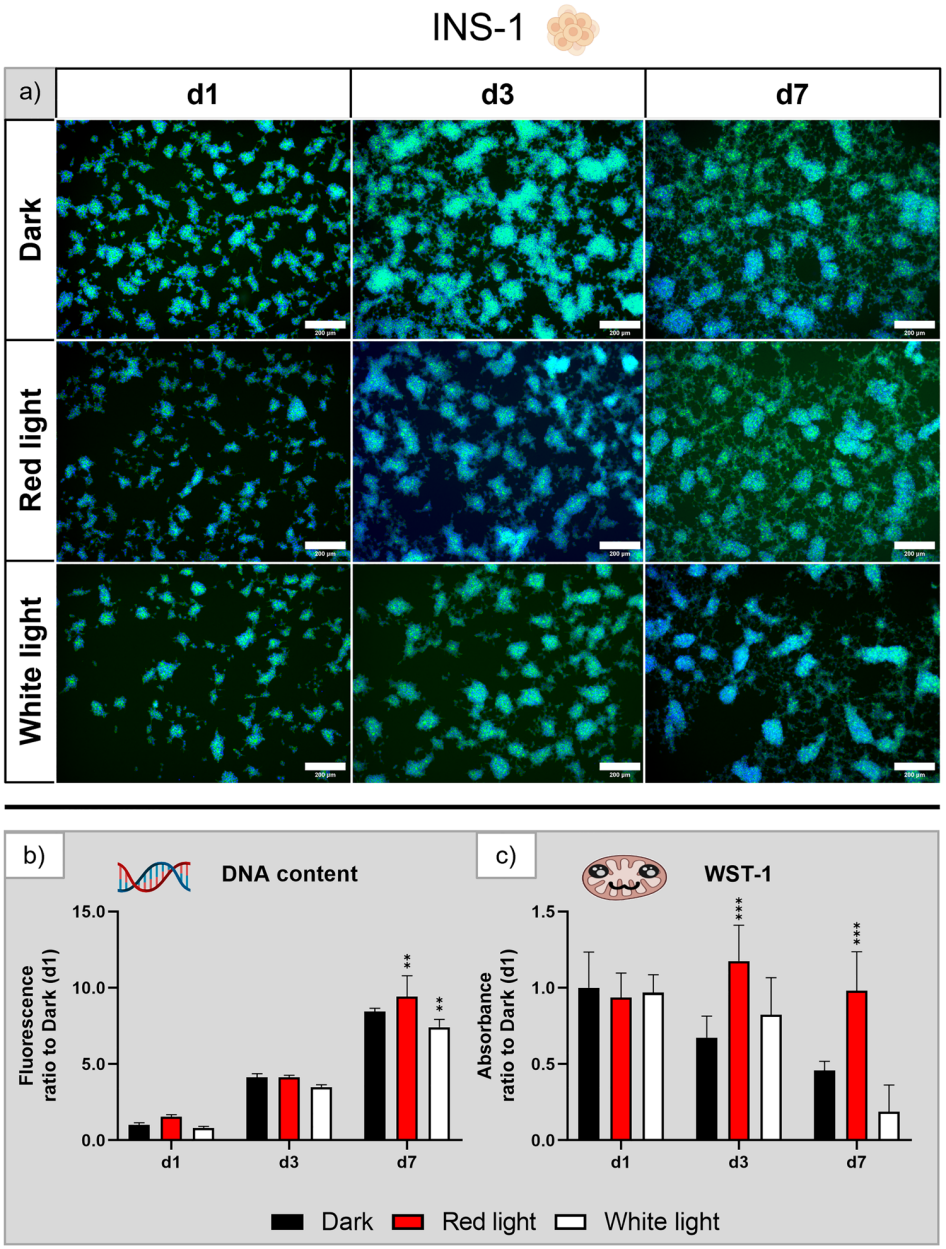
Influence of red and white light illumination on INS-1 during cultivation over seven days: (**a**) microscopy images of fluorescence staining (green—actin cytoskeletons, blue—cell nuclei), scale bar: 200 µm; (**b**) DNA content and (**c**) metabolic activity, both normalised to the dark control on day 1. n = 4, mean ± SD; p-values: **p < 0.01; ***p < 0.001.

Fluorescence microscopy images of the INS-1 cell line (Fig. 5a) revealed no difference between the three groups on day 1 and day 3 of culture; on day 7, the area coverage in the white light group seemed to be slightly reduced compared to the dark and red light groups. Cells in all groups exhibited the clustering characteristic of INS-1 with increasing cluster size over the course of the experiment. The DNA content (Fig. 5b) increased eightfold in the dark control group until day 7. Both illuminated groups showed no significant differences from the reference on day 1 and day 3; on day 7, the DNA content of the red light illuminated group was significantly increased, whereas the white light illuminated cells had a significantly lower DNA content. The WST-1 assay (Fig. 5c) revealed no significant influence of white light illumination on the metabolic activity but a twofold increase for cells under red light illumination on day 3 and day 7.

The repeating experiments (Supplementary Fig. S6) confirmed these findings only partially. Despite of this limitation, it can be stated that the INS-1 showed no morphological changes under red and white light illumination and that there was no clear trend towards a negative or positive effect of red or white light on the metabolic activity. A significantly reduced DNA content after seven days of white light exposure was observed in all three experiments which was supported by the microscopic analysis.

#### Investigation of glutathione levels

**Figure 6:**
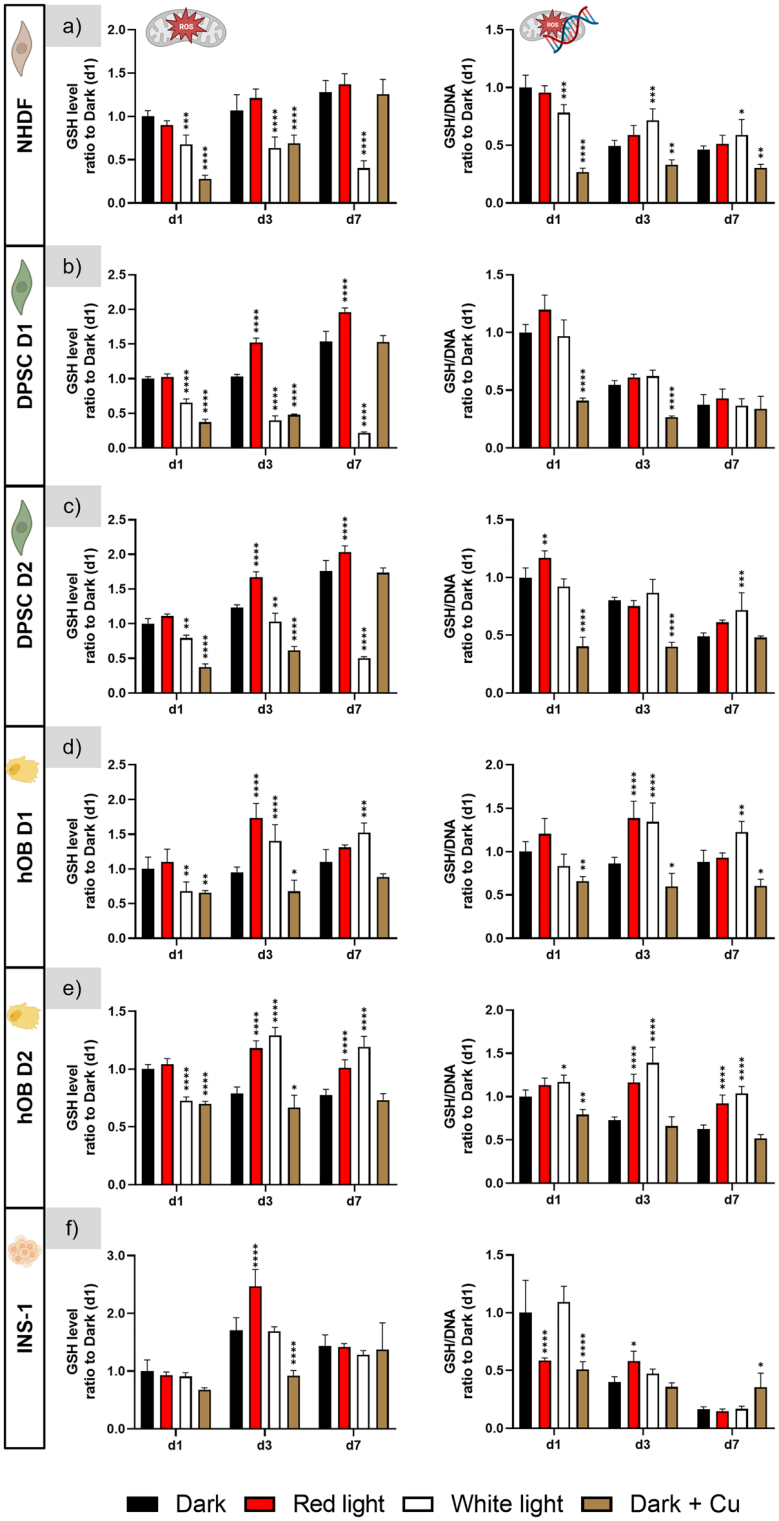
GSH levels of all investigated cell types cultured under red and white light illumination, with a group cultivated in darkness as negative control and a positive control group with added copper ions in the standard cultivation medium. Left side: GSH levels normalised to that measured for the dark control on day 1; right side: ratio of GSH level to DNA content of the same well, then normalised to the ratio determined for the dark control on day 1. n = 4, mean ± SD; p-values: *p < 0.05; **p < 0.01; ***p < 0.001; ****p < 0.0001.

Measurement of the intracellular GSH levels of NHDF revealed up to threefold reduced values on day 1, day 3 and day 7 of cultivation under white light; GSH levels measured for the red light-exposed cells were similar to the dark control. However, when normalized to the respective DNA content, the GSH/DNA values of the white light group were in the same range to those of the dark and the red light groups; even significantly higher on day 3 and day 7 (Fig. 6a).

Similar results were obtained for the DPSC of both donors: Under white light illumination, the measured GSH levels were significantly reduced compared to the dark control group, but GSH levels normalized to the respective DNA were not significantly different to the dark control group. Interestingly, GSH levels were significantly higher under red light compared to the dark control, but these effects were also offset when GSH levels were related to the DNA content (Fig. 6b,c).

For the hOB of both donors, significantly increased GSH levels were measured both under red and white light illumination at day 3 and day 7. This effect was also visible when GSH levels were related to the DNA content. Only on day 1 of cultivation, the GSH level was significantly reduced after white light illumination, but the GSH/DNA value was not significantly lower compared to the dark control group (Fig. 6d,e).

For the INS-1, the MCB assay revealed only a statistically significant increase of the GSH level for cells under red light illumination at day 3, which was also detectable in the GSH/DNA levels. However, also the positive copper control did not show reduced GSH levels at d1 and d7 (Fig. 6f).

The functionality of the assay under the experimental settings of this study was proven by adding copper to the medium of some samples cultivated in darkness as a positive control, since formation of ROS in presence of copper ions and thus lowering the measurable GSH content within the cells is known^33–35^. Compared to the (copper-free) dark control group, a significant reduction of both the GSH level and the GSH/DNA value was observed for NHDF, DPSC, and hOB on day 1 and day 3 of culture in the copper control group. On day 7, the values were in most cases not significantly different to the copper-free control, indicating adaptation of the cells (Fig. 6a–e). For the INS-1, a significant reduction was observed on day 1 (GSH/DNA) and on day 3 (GSH) (Fig. 6f).

Despite of some variations, the two repeating experiments (Supplementary Figs. S7 and S8) confirmed the findings presented in Fig. 8. Thus, the data of this study do not indicate for any of the cell types examined a reduced GSH value when related to the DNA content.

### Influence of long-term blue light illumination

#### Normal human dermal fibroblasts

**Figure 7:**
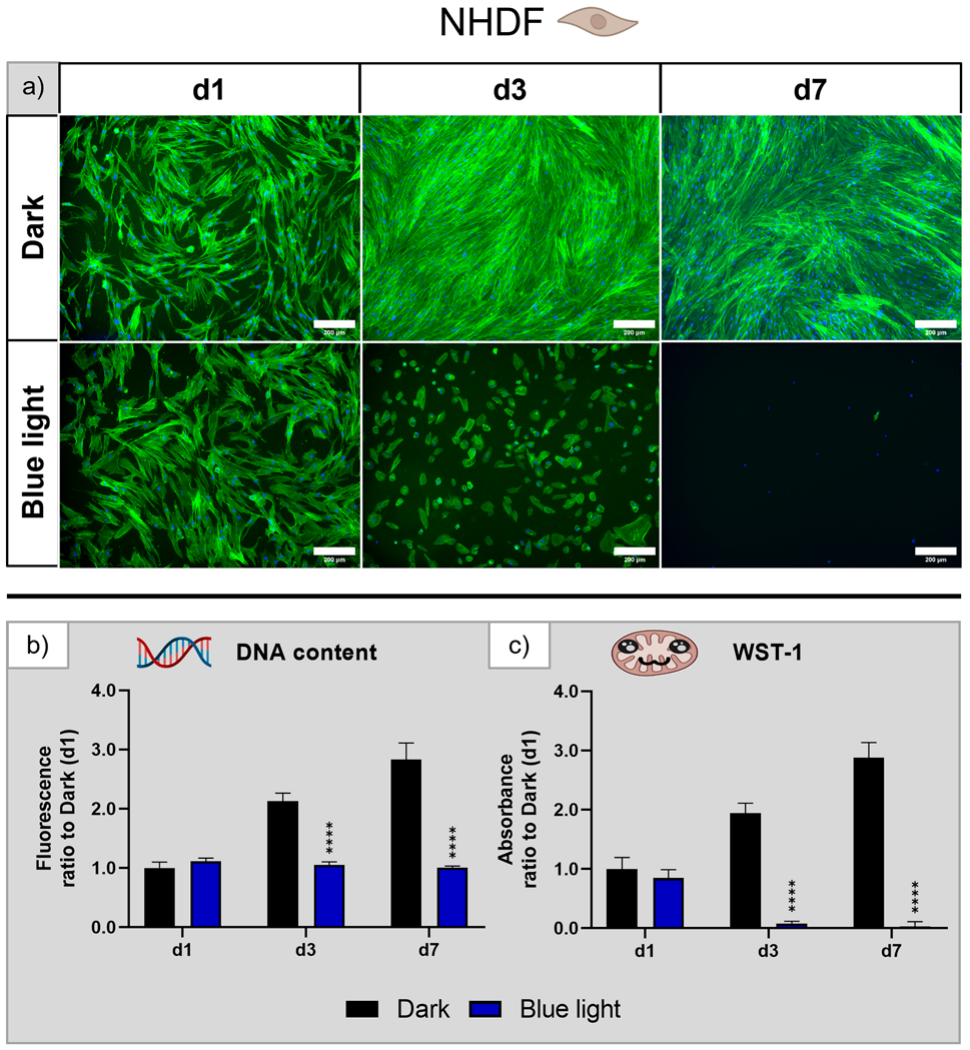
Influence of blue light illumination on NHDF during cultivation over seven days: (**a**) microscopy images of fluorescence staining (green—actin cytoskeletons, blue—cell nuclei), scale bar: 200 µm; (**b**) DNA content and (**c**) metabolic activity, both normalised to that measured for the dark control on day 1. n = 4, mean ± SD; p-values: ****p < 0.0001.

Fluorescence microscopic images (Fig. 7a) indicated a strong influence of blue light illumination on NHDF: While cell density and elongated shape of the cells were very similar to the dark control group on day 1, by day 3 the cells in the blue light condition appeared rounded and the cell number was visibly reduced. By day 7 of the experiment, there were virtually no intact cells in the blue light group, while the cells grown in darkness formed a closed layer. Similarly to the white light condition, the drastic decrease in cell number over the cultivation period is not reflected in the DNA content which was, nevertheless, threefold lower compared to those of the dark control group after three and seven days of culture (Fig. 7b). In accordance with the microscopic images, the WST-1 assay (Fig. 7c) revealed very low metabolic activity of NHDF which were exposed to blue light for three days; after seven days, metabolic activity was barely detectable. Thus, NHDF seem to react similarly to white and blue light, but the effects of blue light are much more pronounced.

#### Human dental pulp stem cells

**Figure 8:**
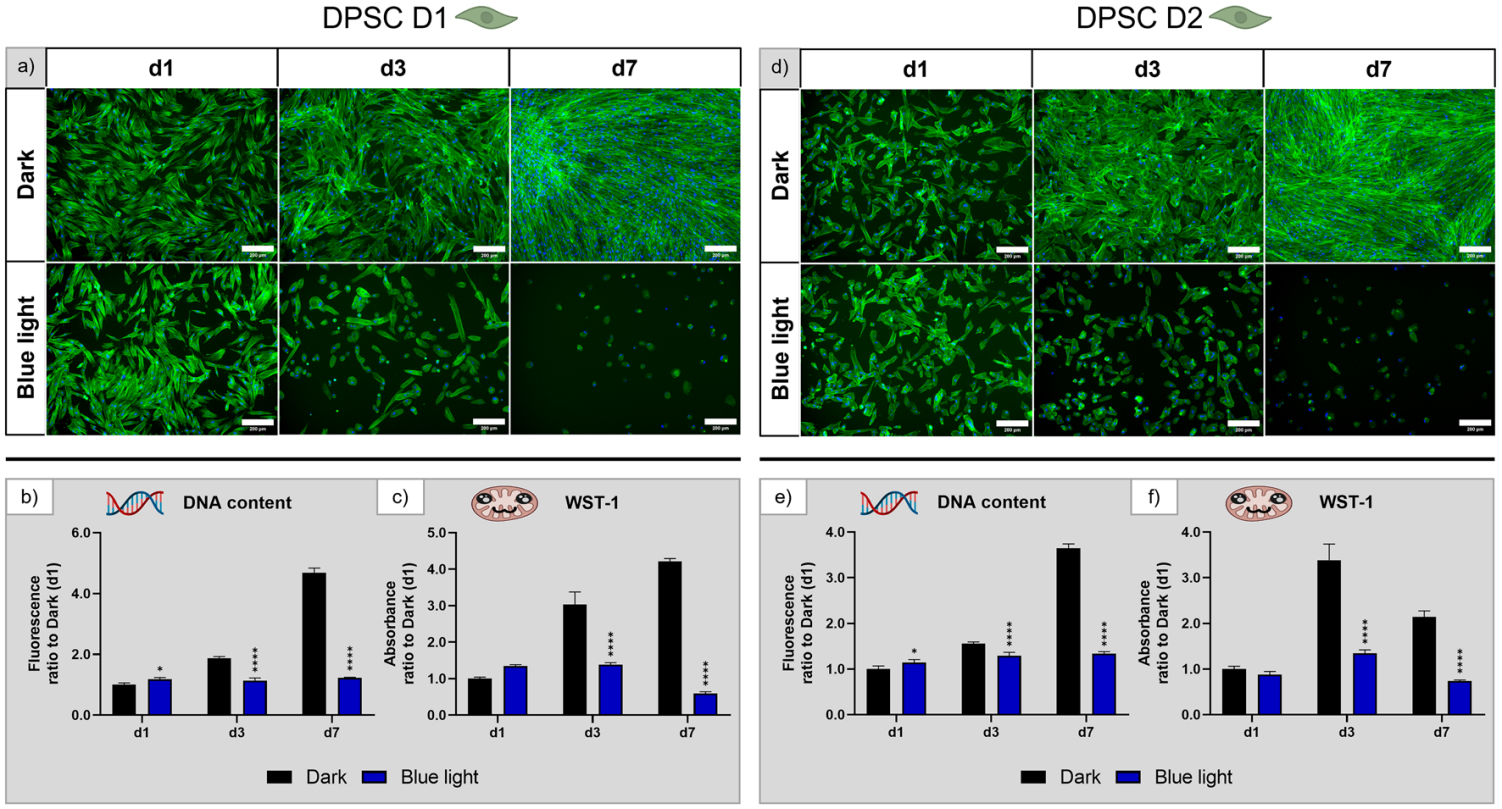
Influence of blue light illumination on DPSC derived from donor 1 (D1) and donor 2 (D2) during cultivation over seven days: (**a**) and (**d**) microscopy images of fluorescence staining (green—actin cytoskeletons, blue—cell nuclei), scale bar: 200 µm; (**b**) and (**e**) DNA content and (**c**) and (**f**) metabolic activity, all normalised to the dark control on day 1. n = 4, mean ± SD; p-values: *p < 0.05; ****p < 0.0001.

Fluorescence microscopic images of human DPSC of both donors (Fig. 8a,d) revealed that the number of attached cells decreased by day 3 and only few attached cells were visible on day 7 under blue light illumination which did not exhibit the typical elongated morphology. Again, the considerably reduced number of attached cells was not entirely reflected by the DNA content (Fig. 8b,e), which was stable over the course of seven days, while the DNA content in the dark control group increased approx. fourfold. Metabolic activity was likewise significantly reduced under blue light exposure (Fig. 8c,f). Thus, blue light has a negative effect on morphology, growth, and metabolic activity of DPSC similar to white light.

#### Human osteoblasts

**Figure 9:**
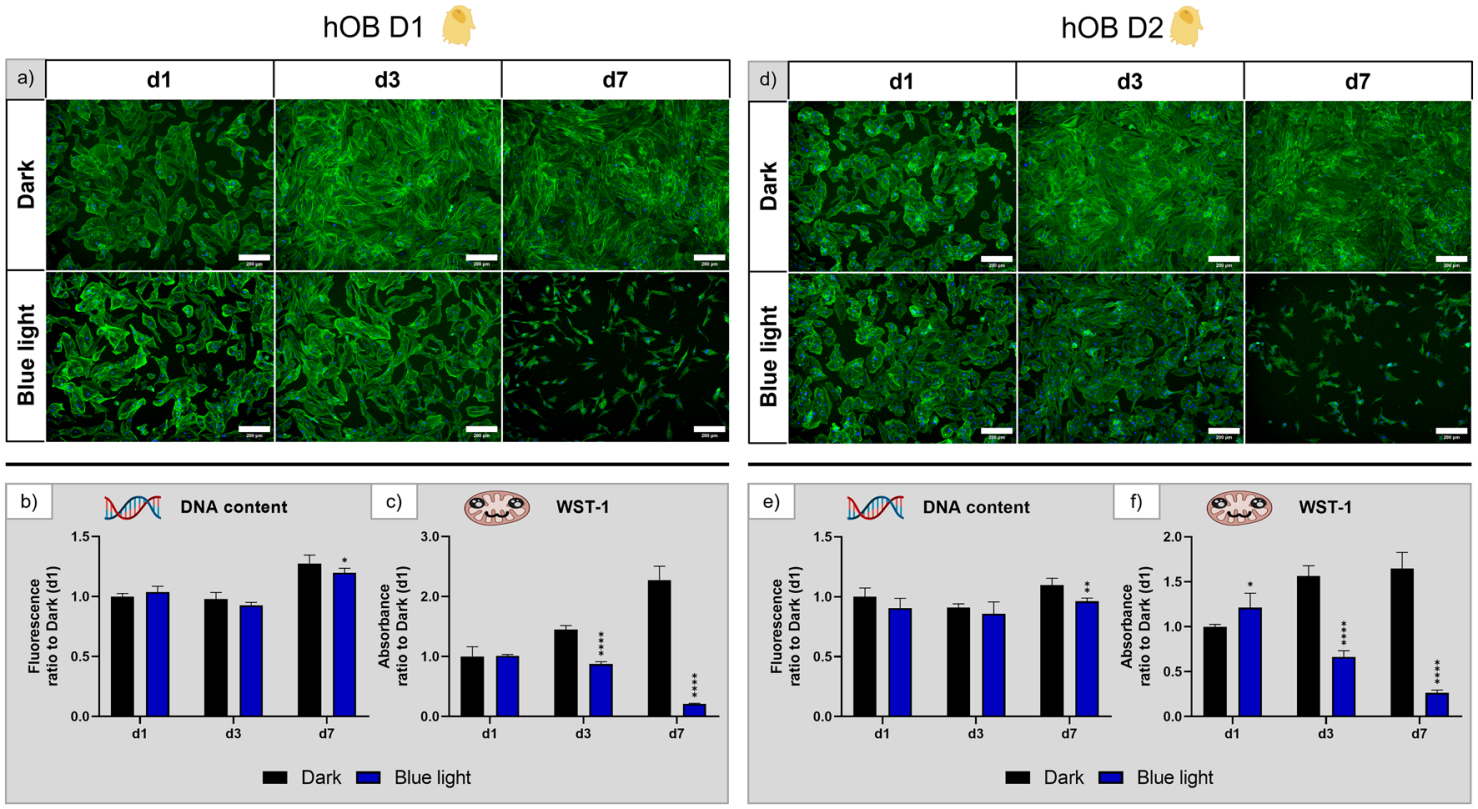
Influence of blue light illumination on hOB derived from donor 1 (D1) and donor 2 (D2) during cultivation over seven days: (**a**) and (**d**) microscopy images of fluorescence staining (green—actin cytoskeletons, blue—cell nuclei), scale bar: 200 µm; (**b**) and (**e**) DNA content and (**c**) and (**f**) metabolic activity, all normalised to the dark control on day 1. n = 4, mean ± SD; p-values: *p < 0.05; **p < 0.01; ****p < 0.0001.

For hOB of the two donors, fluorescence microscopic images (Fig. 9a,d) showed a slight decrease in the density of attached cells by day 3 under blue light illumination, which became much more pronounced after seven days. On day 3, the typical morphology was still retained whereas on day 7, the cells appeared to be less spread and not connected to each other. Accordingly, DNA content was significantly decreased at day 7 under blue light (Fig. 9b,e). In addition, the metabolic activity of the cells was significantly reduced after exposure to blue light for three and seven days (Fig. 9c,f). Thus, blue light has a negative effect on morphology, growth, and metabolic activity of hOB that is more pronounced than the effect of white light.

#### Murine cell line INS-1

**Figure 10:**
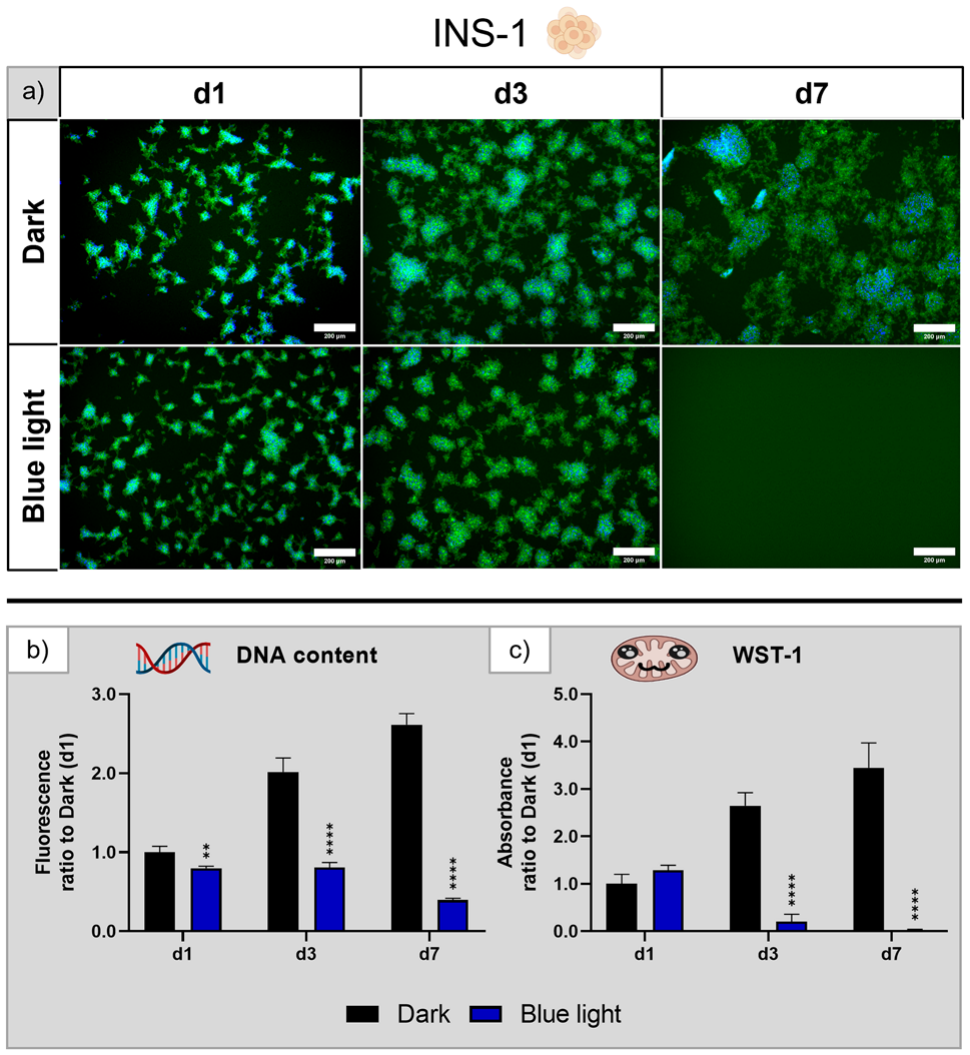
Influence of blue light illumination on INS-1 during cultivation over seven days: (**a**) microscopy images of fluorescence staining (green—actin cytoskeletons, blue—cell nuclei), scale bar: 200 µm; (**b**) DNA content and (**c**) metabolic activity, both normalised to that measured for the dark control on day 1. n = 4, mean ± SD; p-values: **p < 0.01; ****p < 0.0001.

Fluorescence microscopic images of INS-1 (Fig. 10a) revealed that INS-1 developed similarly in darkness and under blue light illumination on day 1 and day 3, with a similar cell number and the characteristic cluster formation. However, on day 7, no cells were detectable in the blue light group while the cells in the dark control group continued to grow and cluster normally. This effect is reflected in the DNA content (Fig. 10b), which was more than twofold lower at all time points under blue light. The metabolic activity (Fig. 10c) of cells exposed to blue light was significantly lower compared to the dark control group at day 3 and barely detectable at day 7. Thus, blue light has a strong negative effect on INS-1 morphology, growth, and metabolic activity.

#### Investigation of glutathione levels

**Figure 11:**
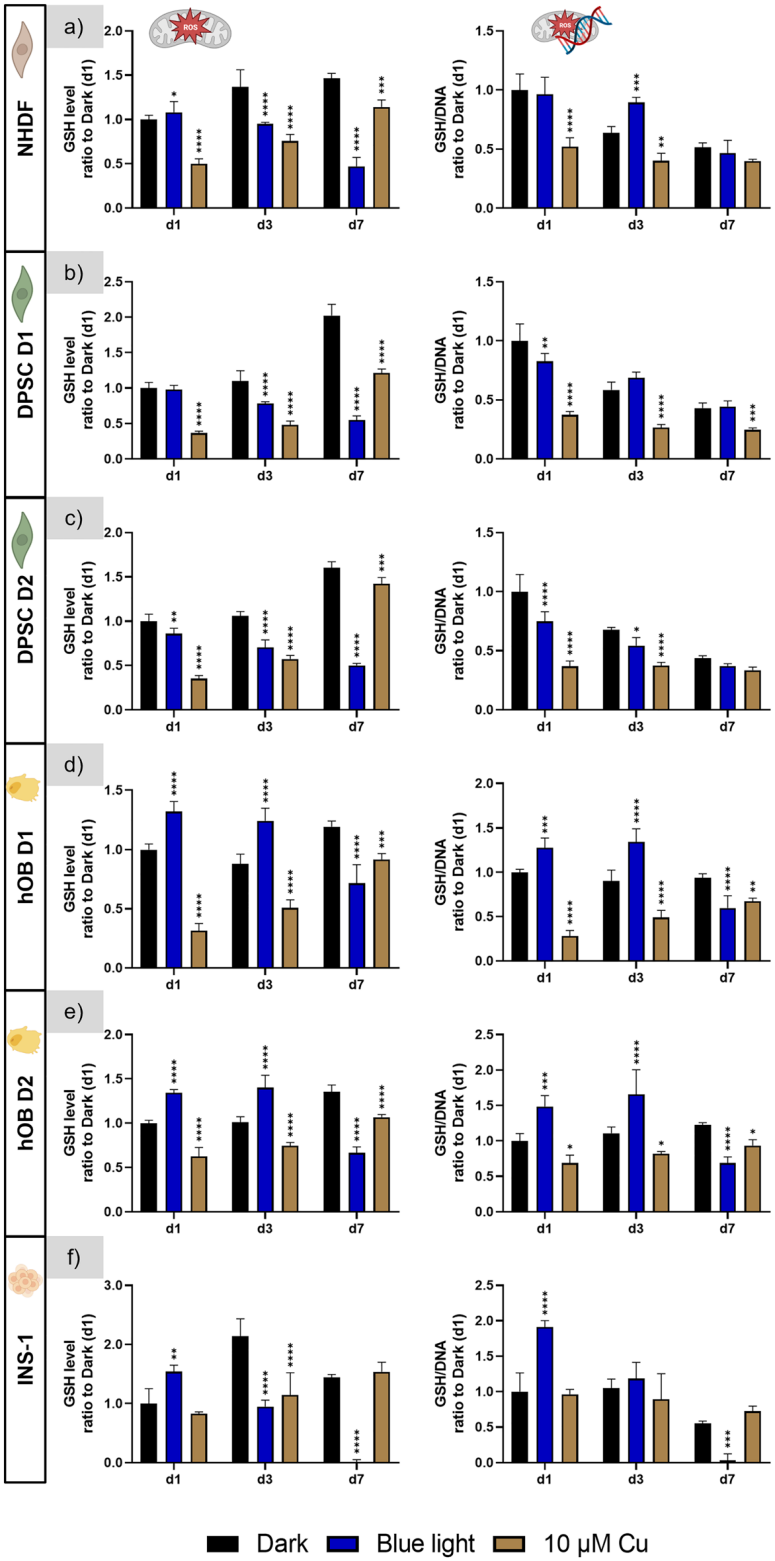
GSH levels of all investigated cell types cultured under blue illumination, with a group cultivated in darkness as negative control and a positive control group with added copper ions in the standard cultivation medium. Left side: GSH levels normalised to that measured for the dark control on day 1; right side: ratio of GSH level to DNA content of the same well, then normalised to the ratio determined for the dark control on day 1. n = 4, mean ± SD; p-values: *p < 0.05; **p < 0.01; ***p < 0.001; ****p < 0.0001.

GSH levels determined for NHDF under blue light illumination were significantly decreased compared to the dark control, however, for GSH related to the DNA, this effect was not detected (Fig. 11a). For DPSC of both donors, significantly decreased GSH levels were measured under blue light compared to the dark control at all examined time points (Fig. 11b,c). Related to the DNA (GSH/DNA), significant lower values were only detected at day 1 as well as at day 3 for D2 (Fig. 11b,c). Interestingly, for hOB of both donors, the GSH levels as well as the GSH/DNA values were significantly increased on day 1 and day 3 of culture under blue light illumination compared to the dark control but significantly decreased on day 7 (Fig. 11d,e). Finally, GSH levels of INS-1 under blue light exposure were significantly reduced on day 3 and day 7, however the GSH/DNA levels were only lowered on day 7 (Fig. 11f).

In summary, the data indicate a reduced GSH value when related to the DNA content only for DPSC (both donors) and hOB (both donors), but not at all investigated time points. Again, all cell types were cultivated in medium with added copper as positive control. The correct application of the assay was indicated by lowered GSH and GSH/DNA levels for the copper group of NHDF, DPSC, and hOB. INS-1 cells seem to react less sensitive to copper and therefore, correct application of the assay could not be clearly proven for this cell type.

### Influence of cell culture medium illuminated with red, white, and blue light

**Figure 12:**
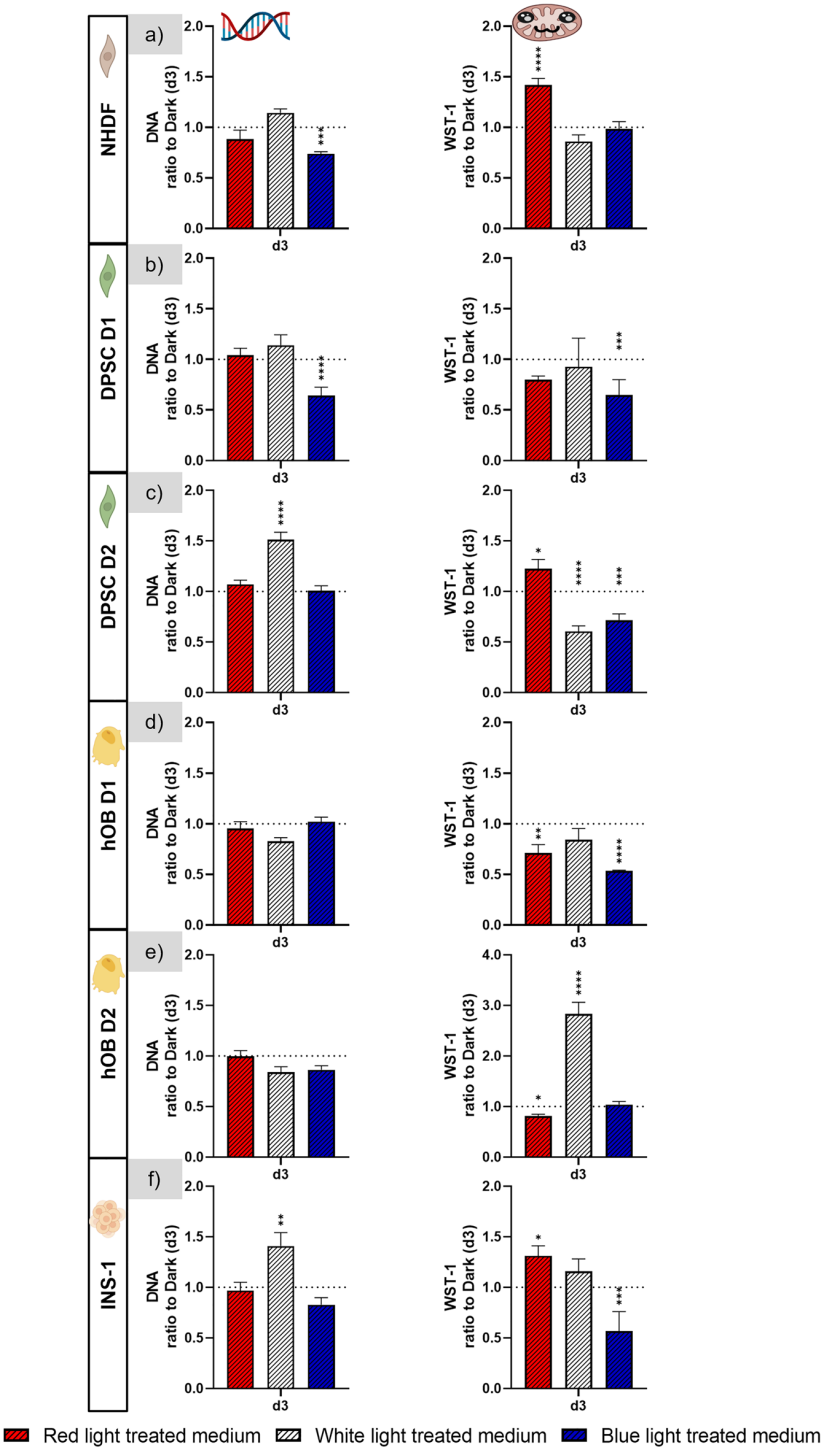
Influence of cell culture medium treated by red, white, or blue illumination on DNA content and metabolic activity of all investigated cell types measured on day 3. The dotted line represents the values of the respective dark control group (medium which was not illuminated) on day 3. n = 4, mean ± SD; p-values: *p < 0.05; **p < 0.01; ***p < 0.001; ****p < 0.0001.

To clarify whether the observed effects of illumination on cell viability and growth are due to a direct influence of light on the cells or caused by light-induced changes of the cell culture medium and its supplements, the cells were cultured in the dark with medium which was pre-illuminated with red, white, or blue light before adding to the cells. Figure 12 shows the data of the DNA quantification and the WST-1 assay of each ‘light-treated medium’ group related to the data of the respective dark control group (without light-exposure of the medium) on day 3 of culture; the data obtained on day 3 are representative for the whole experiment (day 1, 3 and 7). The statistical significance of differences was calculated for comparisons of each ‘light-treated medium’ group with the dark control.

The DNA content was not significantly reduced for any cell type cultivated in red or white light treated medium. In blue light treated medium, the DNA content of NHDF (0.75) and DPSC D1 (0.6) was significantly reduced. The WST-1 assay revealed a significantly decreased metabolic activity of both hOB donors (0.7 and 0.8) in red light treated medium, and of DPSC D2 (0.6) in white light treated medium. In blue light treated medium, the metabolic activity of both DPSC donors D1 (0.65 and 0.7), of hOB D1 (0.55) and of INS-1 (0.6) was significantly reduced. Interestingly, in some cases, a significant increase was observed for media exposed to white and red light but this did not occur in any case in both DNA content and the metabolic activity measurements.

Supplementary figure S9 shows the fluorescence microscopy images on day 3 of the cells exposed to light treated medium and images of the respective dark control group. These data revealed no negative effect of red light treated medium on cell density and morphology of any cell type. The cell density of hOB of both donors as well as DPSC D2 was reduced in white light treated medium, medium exposed to blue light negatively affected the observed cell density of all cell types except for INS-1.

In summary, there was no reproducible negative influence of red or white light on cell culture medium observed. A negative effect of blue light-treated medium on the metabolic activity of cells is possible since a significant reduction was observed in 4 out of 6 experiments.

## Discussion

The influence of illumination on mammalian cells was previously discussed extensively in various contexts. However, in contrast to the studies carried out under the aspects of optogenetics, live cell imaging or photobiomodulation, the proposed co-culture with photosynthetically active microalgae requires a constant illumination in a spectral range between 400 and 700 nm over the span of several days or even weeks. Therefore, the methods and parameters describing the influence of light on mammalian cells for short-term exposure might not be applicable for such a long-term approach.

After reviewing the literature and taking the methods and findings of other studies into account, we selected a range of analyses that are expected to provide insights into the effect of light of different wavelengths on several cell types. By opting for 96-well cell culture plates, quadruplicates of each experimental group were possible while still conforming to the space constrains given by the illumination setup. The parameters of cell density and morphology, obtained by actin cytoskeleton and nuclei staining, cell number, obtained by quantifying the DNA content, and metabolic activity based on mitochondrial dehydrogenases using the WST-1 assay, were selected because they were applicable to all selected cell types without adapting and changing the method. Furthermore, the combination of these analytical methods yielded both mutually confirming and complementary information that finally provided a reliable picture of long-term illumination effects on mammalian cells.

The strength of the illumination effects on the cells varied between the different analytical methods. For example, a drastic decrease in cell density between day 1 and day 7 was observed in fluorescence microscopy for all cell types under white and blue light illumination (except for hOB under white light), which was not reflected with the same intensity in the analysis of the DNA content. Here, the differences in sample processing must be taken into account that may have caused this discrepancy: The high number of washing steps, required in the preparation and staining of the microscopy samples, may have resulted in the detachment also of unhealthy or dying cells. The DNA analysis requires a much lower number of handling steps and therefore may preserve a higher number of non-healthy cells. The presence of non-healthy cells, already affected by illumination, was apparent from an altered morphology of the cells—especially in case of cells illuminated with blue light. Comparison of the data obtained from DNA quantification with the data from the WST-1 assay indicates that in most cases the metabolic activity of the cells is more sensitive to illumination than the cell number derived from DNA content. This was observed especially for cells under blue light illumination. Interestingly, although no obvious effect of illumination with white or blue light on hOB was detected via DNA content and—in case of white light—via microscopic analysis, a significant reduction of the metabolic activity was determined in the WST-1 assay. Therefore, due to the high accuracy and highest sensitivity of the WST-1 assay, the analysis of metabolic activity was chosen as the main parameter to assess the influence of light on the selected cell types. Measurement of metabolic activity was well supported by findings from fluorescence microscopic analysis of morphology and density of the cells.

*Influence of white light.* The continuous illumination with white, sun-like light revealed a negative impact on all investigated cell types, varying in severity depending on the cell type. The strongest response showed NHDF and DPSC since all three analytical methods revealed significant differences to the dark control from day 3 on. DNA content did not increase over the 7 days of illumination in contrast to the increasing DNA content of the dark controls, indicating an early inhibitory effect of white light on growth and activity of these cells. In contrast, hOB seemed to be less affected by white light illumination: For both donors, the findings indicated an early impact on metabolic activity, even though cell morphology and growth are not noticeably impaired. It stands to reason that while hOB react less severely to white light, a negative influence also on cell density and DNA content would possibly be notable at later cultivation time points. The murine cell line INS-1, the only non-human and non-primary cell type in this experiment, showed an aberrant behaviour. Here, only the DNA content is significantly reduced on day 7 under white light illumination, all other values were not significantly different to the dark control. This behaviour might be explained by the characteristic cluster formation of the INS-1^31^, protecting the inner cells from illumination. In line with this assumption is the observation of Mignon et al. that the inhibitory effect of short wavelength light on fibroblasts was reduced with increasing cell confluency^36^. While the observations for DPSC are fully consistent with our previous work (in which cell viability was determined after simultaneous staining of live and dead cells and the cell number was determined by DNA quantification)^27^, the decrease in DNA of INS-1 cells under white light illumination was more pronounced in the present study . A possible reason could be the different light source used in that study; side effects of illumination like a slight increase in temperature in the cell culture plates can hardly be recorded and therefore not excluded. All in all, the findings of the present study suggest a strong influence of white light specifically on the metabolic activity of cells, which presents a change at earlier time points than DNA content and cell morphology. Thus, the stronger proliferating NHDF and DPSC, that are in turn more metabolically active are more heavily impacted by white light illumination than the less proliferating and therefore less active hOB.

White light covers the entire spectrum of visible light, with peaks of intensity in the blue and red spectral ranges. To test the hypothesis of our study, cells were treated with red and blue light to investigate whether the long-wave red light does not affect mammalian cells^37^ even under continuous, long-term illumination and whether the phototoxic effect of white light is caused by its blue light component.

*Influence of red light*. Red light covers the longest wavelength portion of the visible light spectrum, which has the least energy. Our previous study and literature about short-term light exposure have shown that red light does not have any negative effects on the growth and functionality of mammalian cells^5,9,27,36^. In the research field of photobiomodulation, there are even studies reporting a positive or stimulatory effect of cyclic short-term exposure to red light on cell viability, proliferation, differentiation, or secretion of signalling factors^11–13^. None of the cell types examined in this study showed any negative effect of red light illumination on cell density or morphology: The fluorescence images from all days remained similar to those of the respective dark controls. The DNA content of red light-illuminated NHDF, DPSC, and INS-1 increased significantly throughout the experiment and remained on similar levels compared to the dark control —in some cases even exceeding those of the control group. The metabolic activity of NHDF, DPSC, and INS-1 showed a similar trend, with comparable or even significantly higher levels of activity under red light illumination compared to darkness. The slower growing hOB exhibited comparable DNA content in the red light and dark control groups; the metabolic activity of red light-treated cells was in the same range as that of the dark control group. However, the results for the hOB were more inconsistent: both assays, DNA quantification and WST-1, showed a few significantly increased or decreased values of the red light group in comparison to the dark control group—no positive trend of red light illumination was observed as with the other cell types tested. Nevertheless, the data clearly indicate that illuminating hOB with red light had no harmful impact.

*Influence of blue light and ROS formation*. By investigating the effect of blue light on the mammalian cell types, we wanted to confirm that the high-energy, short-wavelength component of white light is responsible for its phototoxic effect and that it is associated with the formation of ROS as described in the literature^3,28^. Indeed, the data indicate a strong negative influence of blue light on all cell types that is more pronounced than that of white light. Even for the hOB and the INS-1, not only the WST-1 assay revealed a significant reduction in metabolic activity but also the microscopic analysis demonstrated a dramatic reduction in cell number over the cultivation time. The stronger impact of blue light in comparison to white light can be explained by the smaller portion and lower intensity of blue light within the white light spectrum: In white light, the intensity peak of blue light is lower compared to red light, while under blue light illumination, a higher intensity of short-wave light is present.

Since one of the primary effects of photons in mammalian cells is an increase of the ROS level ^3,15^, we aimed to identify potential links between ROS levels and DNA content as well as metabolic activity under illumination with the three different light qualities. To this end, we applied the MCB assay to determine the intracellular GSH levels as a means of indirectly quantifying the amount of ROS as recently described for human osteoclasts and their precursors^33^. The GSH level was also determined in another study investigating the effect of blue light illumination on rat osteoblasts, where illumination led to lowered GSH levels^28^. However, the experimental setup was very different compared to this study: Cells were illuminated only for 3 h while submerged in buffer solution, while here we illuminated cells continuously for up to 7 days in medium containing ROS scavengers like sodium pyruvate. Therefore, in order to prove that the experimental conditions used in the present study did not impede the detection of ROS formation, a positive control group was incorporated for each cell type, where copper ions were added to the medium. In preliminary experiments, the optimal concentration of copper ions was determined for every cell type, where ideally the GSH levels were decreased, indicating ROS formation, but the DNA content was not affected. The results of the positive control group were deliberately included in the figures to demonstrate the assay’s functionality, showing that at least on day 1 and day 3, both GSH levels and normalised GSH levels (related to the DNA content) were decreased for the positive control for all cell types except for the murine INS-1 cell line. Represented over all three experiments with red and white light illumination, no clear indication could be found that INS-1 reacted to the supplied amount of copper in the desired way. Therefore, the results were again inconclusive: While there are significant differences for white or red light illumination in some experiments and on some days, there is no trend visible that is a limitation of this experiment.

To depict cell stress, normalising the measured GSH levels to the DNA content is a reasonable approach, since GSH levels are dependent on the cell number of a sample. This was especially important since some cell types exhibited a drastic decrease in DNA content as a reaction to light exposure—in this case, a decrease in GSH level could be a result of an increased ROS level, a decreased cell number or both. However, since in literature often only the GSH level (not related to the cell number/DNA content) is depicted^28,32^, we decided to show both the measured GSH levels and the GSH/DNA ratios. In all cell types, ROS formation appeared to be not influenced by red light, indicated by the GSH level and by the GSH/DNA ratio. Exposure to white light reduced the total amount of intracellular GSH for NHDF, DPSC and partially hOB. However, a simultaneous decrease in DNA content cancelled out this effect and thus, excluded ROS formation as (only) reason for the phototoxic effect of continuous white light illumination. Also, for blue light illuminated cells, a clear correlation of the cytotoxic effects and an enhanced ROS formation were not observed: A reduced GSH/DNA value was only found at some time points for DPSC and hOB.

These results are in contrast to reports in literature, where a correlation between high doses of light and reduced cell viability was explained by increased ROS formation e. g. for primary human fibroblasts^38^, and rat osteoblasts^28^. In most studies investigating the effects of illumination on cells, light doses between 5 to 55 J/cm^2^ were applied^28,38,39^.

Ramakrishnan et al.^28^ achieved a higher irradiance with blue light—5 mW/cm^2^ at 405 nm compared to 2.3 mW/cm^2^ at 460 nm in this study—but the cells were only irradiated for 3 h, resulting in a light dose of 54 J/cm^2^. Due to its time-dependency, the light dose applied in this study (after 7 days of illumination) is at least 30 times higher. This highlights the greatest difference between this study and the literature: Usually, the effects of light after a few hours of illumination are described, an increase in ROS is already observable after one hour. In bacteria, a return of ROS concentration to similar levels to the positive control was described after six hours of illumination^28^. Therefore, we cannot exclude that the 24 h of cultivation in this study before the first measurement was too long and shorter intervals are necessary to prove a light dependent increase of ROS. In addition, the cultivation length of seven days made cultivation in cell culture medium imperative. Sodium pyruvate, a component of cell culture medium, was shown to counteract cytotoxic effects of ROS^40^, an effect that was also reported by Ramakrishnan et al. under blue light illumination, who therefore cultivated the cells in buffer solution^28^.

*Indirect influence of light *via* changes in cell culture media*. In order to investigate whether the cytotoxic effect of light is indirectly caused by light-induced changes in the cell culture media, we cultivated the cells in the dark in medium that was pretreated with light. Our data suggest that illumination with red or white light does not lead to significant changes in the cell culture media by photochemical conversion of light-sensitive components that cause cytotoxic or inhibitory effects. However, in the case of blue light illumination it cannot be ruled out that the observed cytotoxic effect is partially mediated via cytotoxic or inhibitory effects of the illuminated media. Most of the studies investigating the cytotoxic effect of light do not distinguish between a direct cytotoxic effect on the cells and a medium-mediated effect. For primary cells of the central nervous system, it was shown that cytotoxic effects of blue light exposure can be prevented by using photostable media and antioxidant-rich, serum-free supplements^8,41^; the development of such media on the basis of DMEM and neurobasal formulations were described in detail by Stockley and coworkers^41^. Mignon et al. compared direct light treatment of primary human dermal fibroblasts with blue light (450 nm) with indirect treatment via irradiated DMEM-based cell culture medium: At lower doses (10 and 30 J/cm^2^), the reduction of metabolic activity was significantly stronger in the directly treated group than in the “pretreated medium”-group but at a higher dose (60 J/cm^2^), both groups showed a similar reduction^36^. This is line with the observation of our study: While the white light group with a lower blue light intensity showed a negligible impact of light pre-treated medium on metabolic activity of the cells, the blue light group showed a more visible response.

For photobiomodulation, it has been described that visible and (near-)infrared light can modulate the cell metabolism without causing significant thermal effects—photons can transfer their energy to molecules in the cell which have retained photoacceptive properties originally developed during earlier phases of evolution^15^. The most important primary target are the mitochondria with the respiratory electron transport chain and therefore, photobiomodulation primarily effects besides the ROS content also the ATP level. Some studies correlated the impact of light on cell metabolism with the responsiveness of the Cytochrome C oxidase, a key component of the mitochondrial electron transport chain and one of the most important photoacceptors of visible light involved in photobiomodulation^39,42^. A clear dependence of the light-induced effect on the energy input was described in many studies; while high-energy (particularly short-wavelength) light causes phototoxicity, less energetic, long-wavelength light can result in an increase of the metabolic activity—as also observed in the present study for red light illumination—e. g. via the mitochondrial electron transport chain.

## Conclusion

Using four different types of mammalian cells, we demonstrated in this study that continuous long-term exposure to red light has no adverse effects on viability, metabolic activity and growth whereas continuous long-term exposure to white light has deleterious effects. This can be explained by the blue light portion of white light. The cytotoxic effects of short-wavelength light observed in our study is predominantly a direct effect of cell illumination, however, a contribution of an indirect effect via light-induced changes in the cell culture media cannot be excluded. A clear correlation of the cytotoxic effect of short-wavelength light with an increased ROS level is not indicated by our data. Further studies are required to analyze ROS formation by an alternative method.

Looking at the literature, it is obvious that there is a huge variance in experimental conditions and analyzed parameters between different studies that might be the reason for (partly) contradictory results and the difficulty in obtaining a conclusive picture of the effect of light on mammalian cells. Besides the illumination parameters (wavelength, intensity, frequency and duration, dose), the cultivation parameters (e. g. serum concentration, photo-sensitive/stable medium components, cell confluency, medium refreshment, oxygen concentration) have a significant influence on the light-induced effects in mammalian cells^36,41^. Also, different cell types show a different sensitivity^37^ that is confirmed by our study, too.

Continuous long-term illumination with red-light is compatible with mammalian cells and can be applied for bio-printed, patterned coculture constructs of mammalian cells with microalgae; red light is also applicable as light source for the microalgae to enable photosynthetic oxygen production^27^.

## Supplementary Information

Supplementary Figures.

## Data Availability

The datasets generated during the current study are available from the corresponding author on reasonable request.

## References

[1] Mansouri, M., Strittmatter, T. & Fussenegger, M. Light-controlled mammalian cells and their therapeutic applications in synthetic biology. *Adv. Sci.* **6**, 1800952 (2019). 10.1002/advs.201800952 PMC6325585 30643713

[2] Emiliani, V. *et al.* Optogenetics for light control of biological systems. *Nat. Rev. Methods Primers* **2**, 55 (2022). 37933248 10.1038/s43586-022-00136-4 PMC10627578

[3] Icha, J., Weber, M., Waters, J. C. & Norden, C. Phototoxicity in live fluorescence microscopy, and how to avoid it. *BioEssays* **39**, 1700003 (2017). 10.1002/bies.201700003 28749075

[4] Alghamdi, R. A., Exposito-Rodriguez, M., Mullineaux, P. M., Brooke, G. N. & Laissue, P. P. Assessing phototoxicity in a mammalian cell line: How low levels of blue light affect motility in PC3 cells. *Front. Cell Dev. Biol.* **9**, 738786 (2021). 34977004 10.3389/fcell.2021.738786 PMC8718804

[5] Schneckenburger, H. *et al.* Light exposure and cell viability in fluorescence microscopy. *J. Microsc.* **245**, 311–318 (2012). 22126439 10.1111/j.1365-2818.2011.03576.x

[6] Repina, N. A., McClave, T., Bao, X., Kane, R. S. & Schaffer, D. V. *Engineered Illumination Devices for Optogenetic Control of Cellular Signaling Dynamics*. 10.1101/675892 (2019) 10.1016/j.celrep.2020.107737 PMC9357365 32521262

[7] Gorgidze, L. A., Oshemkova, S. A. & Vorobjev, I. A. Blue light inhibits mitosis in tissue culture cells. *Biosci. Rep.* **18**, 215–224 (1998). 9877234 10.1023/a:1020104914726

[8] Duke, C. G., Savell, K. E., Tuscher, J. J., Phillips, R. A. & Day, J. J. Blue light-induced gene expression alterations in cultured neurons are the result of phototoxic interactions with neuronal culture media. *eNeuro* **7**, ENEURO.0386-19.2019 (2020). 10.1523/ENEURO.0386-19.2019 PMC6946540 31879366

[9] Wäldchen, S., Lehmann, J., Klein, T., Van De Linde, S. & Sauer, M. Light-induced cell damage in live-cell super-resolution microscopy. *Sci. Rep.* **5**, 15348 (2015). 26481189 10.1038/srep15348 PMC4611486

[10] Dompe, C. *et al.* Photobiomodulation—Underlying mechanism and clinical applications. *JCM* **9**, 1724 (2020). 32503238 10.3390/jcm9061724 PMC7356229

[11] Yin, K., Zhu, R., Wang, S. & Zhao, R. C. Low-level laser effect on proliferation, migration, and antiapoptosis of mesenchymal stem cells. *Stem Cells Dev.* **26**, 762–775 (2017). 28178868 10.1089/scd.2016.0332

[12] Tani, A. *et al.* Red (635 nm), near-infrared (808 nm) and violet-blue (405 nm) photobiomodulation potentiality on human osteoblasts and mesenchymal stromal cells: a morphological and molecular in vitro study. *IJMS* **19**, 1946 (2018). 29970828 10.3390/ijms19071946 PMC6073131

[13] Kocherova, I. *et al.* Photobiomodulation with red and near-infrared light improves viability and modulates expression of mesenchymal and apoptotic-related markers in human gingival fibroblasts. *Materials* **14**, 3427 (2021). 34205573 10.3390/ma14123427 PMC8233986

[14] Agas, D. *et al.* Photobiomodulation by near-infrared 980-nm wavelengths regulates pre-osteoblast proliferation and viability through the PI3K/Akt/Bcl-2 pathway. *IJMS* **22**, 7586 (2021). 34299204 10.3390/ijms22147586 PMC8304212

[15] Amaroli, A. *et al.* Steering the multipotent mesenchymal cells towards an anti-inflammatory and osteogenic bias via photobiomodulation therapy: How to kill two birds with one stone. *J. Tissue Eng.* **13**, 204173142211101 (2022). 10.1177/20417314221110192 PMC9272199 35832724

[16] Prado, T. P. *et al.* Photobiomodulation with blue light on wound healing: A scoping review. *Life* **13**, 575 (2023). 36836932 10.3390/life13020575 PMC9959862

[17] Wang, Y., Huang, Y.-Y., Wang, Y., Lyu, P. & Hamblin, M. R. Photobiomodulation (blue and green light) encourages osteoblastic-differentiation of human adipose-derived stem cells: Role of intracellular calcium and light-gated ion channels. *Sci. Rep.* **6**, 33719 (2016). 27650508 10.1038/srep33719 PMC5030629

[18] Bloch, K. *et al.* Photosynthetic oxygen generator for bioartificial pancreas. *Tissue Eng.* **66**, 60 (2006). 10.1089/ten.2006.12.337 16548692

[19] Bloch, K. *et al.* Immobilized microalgal cells as an oxygen supply system for encapsulated pancreatic islets: A feasibility study. *Artif. Organs* **30**, 715–718 (2006). 16934101 10.1111/j.1525-1594.2006.00289.x

[20] Evron, Y. *et al.* Oxygen supply by photosynthesis to an implantable islet cell device. *Horm. Metab. Res.* **47**, 24–30 (2014). 25365509 10.1055/s-0034-1394375

[21] Obaíd, M. L. *et al.* A first in human trial implanting microalgae shows safety of photosynthetic therapy for the effective treatment of full thickness skin wounds. *Front. Med.* **8**, 772324 (2021). 10.3389/fmed.2021.772324 PMC8669306 34917636

[22] Hopfner, U. *et al.* Development of photosynthetic biomaterials for in vitro tissue engineering. *Acta Biomater.* **10**, 2712–2717 (2014). 24406198 10.1016/j.actbio.2013.12.055

[23] Lode, A. *et al.* Green bioprinting: Fabrication of photosynthetic algae-laden hydrogel scaffolds for biotechnological and medical applications. *Eng. Life Sci.* **15**, 177–183 (2015).

[24] Trampe, E. *et al.* Functionalized bioink with optical sensor nanoparticles for O_2_ imaging in 3D-bioprinted constructs. *Adv. Funct. Mater.* **28**, 1804411 (2018).

[25] Maharjan, S. *et al.* Symbiotic photosynthetic oxygenation within 3D-bioprinted vascularized tissues. *Matter* **4**, 217–240 (2021). 33718864 10.1016/j.matt.2020.10.022 PMC7945990

[26] Hwangbo, H. *et al.* Photosynthetic cyanobacteria can clearly induce efficient muscle tissue regeneration of bioprinted cell-constructs. *Adv. Funct. Materials.* **33**, 2209157 (2023).

[27] Dani, S. *et al.* Selection of a suitable photosynthetically active microalgae strain for the co-cultivation with mammalian cells. *Front. Bioeng. Biotechnol.* **10**, 994134 (2022). 36199362 10.3389/fbioe.2022.994134 PMC9528974

[28] Ramakrishnan, P., Maclean, M., MacGregor, S. J., Anderson, J. G. & Grant, M. H. Cytotoxic responses to 405 nm light exposure in mammalian and bacterial cells: Involvement of reactive oxygen species. *Toxicol. In Vitro* **33**, 54–62 (2016). 26916085 10.1016/j.tiv.2016.02.011

[29] Neunzehn, J. *et al.* Dentin-like tissue formation and biomineralization by multicellular human pulp cell spheres in vitro. *Head Face Med.* **10**, 25 (2014). 24946771 10.1186/1746-160X-10-25 PMC4074584

[30] Skottke, J., Gelinsky, M. & Bernhardt, A. In vitro co-culture model of primary human osteoblasts and osteocytes in collagen gels. *Int. J. Mol. Sci.* **20**, 1998 (2019). 31018582 10.3390/ijms20081998 PMC6514924

[31] Asfari, M. *et al.* Establishment of 2-mercaptoethanol-dependent differentiated insulin-secreting cell lines. *Endocrinology* **130**, 167–178 (1992). 1370150 10.1210/endo.130.1.1370150

[32] Čapek, J., Hauschke, M., Brůčková, L. & Roušar, T. Comparison of glutathione levels measured using optimized monochlorobimane assay with those from ortho-phthalaldehyde assay in intact cells. *J. Pharmacol. Toxicol. Methods* **88**, 40–45 (2017). 28642085 10.1016/j.vascn.2017.06.001

[33] Bernhardt, A., Bacova, J., Gbureck, U. & Gelinsky, M. Influence of Cu^2+^ on osteoclast formation and activity in vitro. *IJMS* **22**, 2451 (2021). 33671069 10.3390/ijms22052451 PMC7957576

[34] Manzl, C., Enrich, J., Ebner, H., Dallinger, R. & Krumschnabel, G. Copper-induced formation of reactive oxygen species causes cell death and disruption of calcium homeostasis in trout hepatocytes. *Toxicology* **196**, 57–64 (2004). 15036756 10.1016/j.tox.2003.11.001

[35] Angelé-Martínez, C., Goodman, C. & Brumaghim, J. Metal-mediated DNA damage and cell death: Mechanisms, detection methods, and cellular consequences. *Metallomics* **6**, 1358–1381 (2014). 24788233 10.1039/c4mt00057a

[36] Mignon, C., Uzunbajakava, N. E., Raafs, B., Botchkareva, N. V. & Tobin, D. J. Photobiomodulation of human dermal fibroblasts in vitro: decisive role of cell culture conditions and treatment protocols on experimental outcome. *Sci. Rep.* **7**, 2797 (2017). 28584230 10.1038/s41598-017-02802-0 PMC5459822

[37] Kleinlogel, S., Vogl, C., Jeschke, M., Neef, J. & Moser, T. Emerging approaches for restoration of hearing and vision. *Physiol. Rev.*10.1152/physrev.00035.2019 (2020). 32191560 10.1152/physrev.00035.2019

[38] George, S., Hamblin, M. R. & Abrahamse, H. Effect of red light and near infrared laser on the generation of reactive oxygen species in primary dermal fibroblasts. *J. Photochem. Photobiol. B Biol.* **188**, 60–68 (2018). 10.1016/j.jphotobiol.2018.09.004 PMC6214457 30216761

[39] Magni, G. *et al.* Experimental study on blue light interaction with human keloid-derived fibroblasts. *Biomedicines* **8**, 573 (2020). 33291338 10.3390/biomedicines8120573 PMC7762279

[40] Wang, X. *et al.* Pyruvate protects mitochondria from oxidative stress in human neuroblastoma SK–N–SH cells. *Brain Res.* **1132**, 1–9 (2007). 17174285 10.1016/j.brainres.2006.11.032 PMC1853247

[41] Stockley, J. H. *et al.* Surpassing light-induced cell damage in vitro with novel cell culture media. *Sci. Rep.* **7**, 849 (2017). 28405003 10.1038/s41598-017-00829-x PMC5429800

[42] Rossi, F. *et al.* Photobiomodulation of human fibroblasts and keratinocytes with blue light: Implications in wound healing. *Biomedicines* **9**, 41 (2021). 33466557 10.3390/biomedicines9010041 PMC7824830

