# Supplementary material for: The effect of continuous long-term illumination with visible light in different spectral ranges on mammalian cells
Source: Sci Rep. 2024 Apr 24;14:9444. doi: 10.1038/s41598-024-60014-9 (PMC11043379; doi:10.1038/s41598-024-60014-9)
Supplement: Supplementary file 1 — Supplementary Figures. [file 41598_2024_60014_MOESM1_ESM.pdf]

Blinded by the lights: The effect of continuous long-term illumination with visible light in different spectral ranges on mammalian cells

Supplementary material

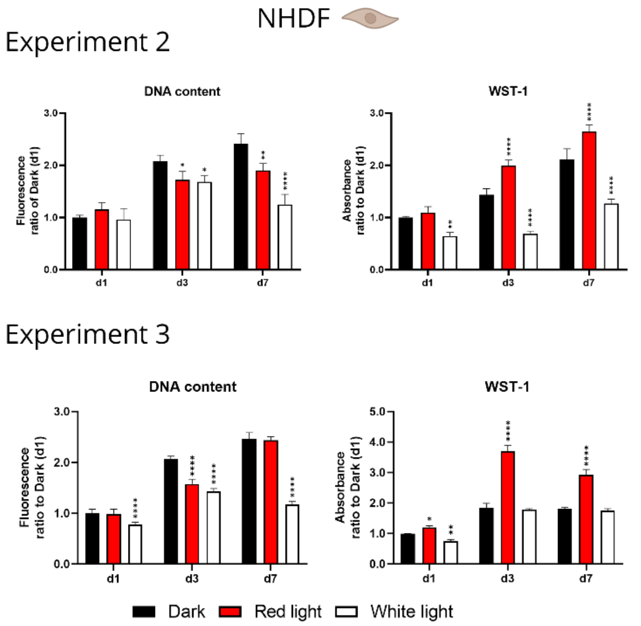

*Supplementary figure S1: Influence of red and white light illumination on NHDF during cultivation over seven days – DNA content and metabolic activity, both normalised to the dark control on day 1, measured in two repeating experiments (experiments 2 and 3); n = 4, mean ± SD; p-values: *p < 0.05; **p < 0.01; ****p < 0.0001.*

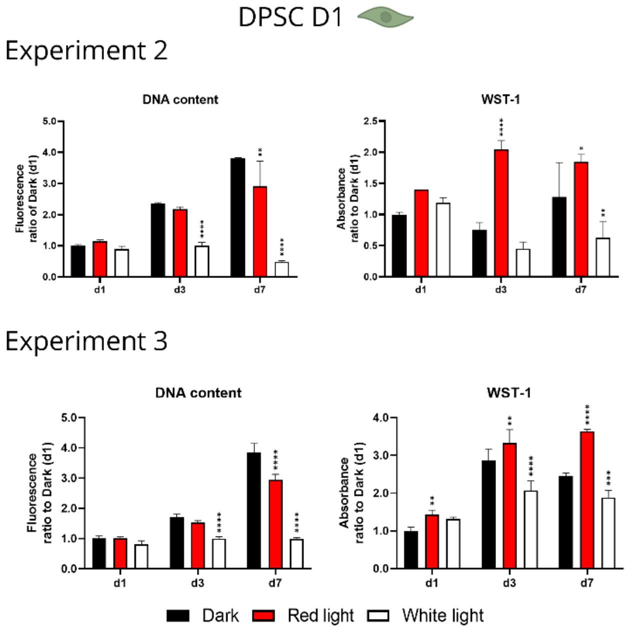

*Supplementary figure S2: Influence of red and white light illumination on DPSC derived from donor 1 (D1) during cultivation over seven days – DNA content and metabolic activity, both normalised to the dark control on day 1, measured in two repeating experiments (experiments 2 and 3); n = 4, mean ± SD; p-values: *p < 0.05; **p < 0.01; ***p < 0.001; ****p < 0.0001.*

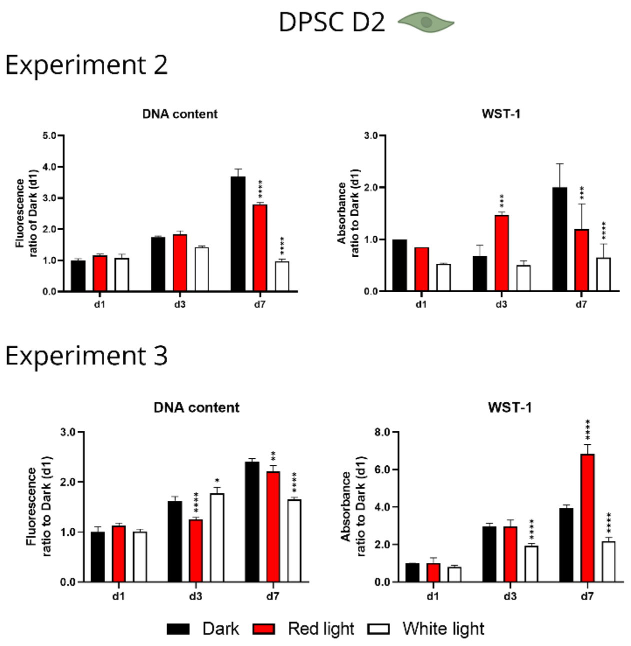

*Supplementary figure S3: Influence of red and white light illumination on DPSC derived from donor 2 (D2) during cultivation over seven days – DNA content and metabolic activity, both normalised to the dark control on day 1, measured in two repeating experiments (experiments 2 and 3); n = 4, mean ± SD; p-values: *p < 0.05; **p < 0.01; ***p < 0.001; ****p < 0.0001.*

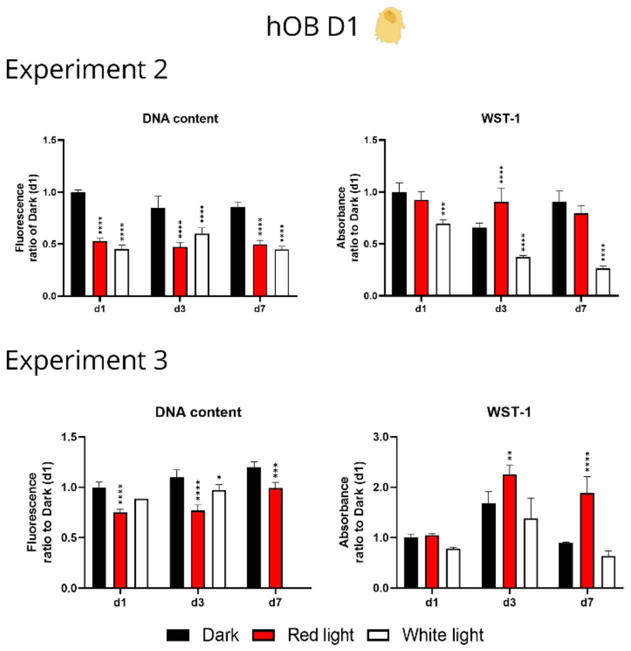

*Supplementary figure S4: Influence of red and white light illumination on hOB derived from donor 1 (D1) during cultivation over seven days – DNA content and metabolic activity, both normalised to the dark control on day 1, measured in two repeating experiments (experiments 2 and 3); n = 4, mean ± SD; p-values: *p < 0.05; **p < 0.01; ***p < 0.001; ****p < 0.0001.*

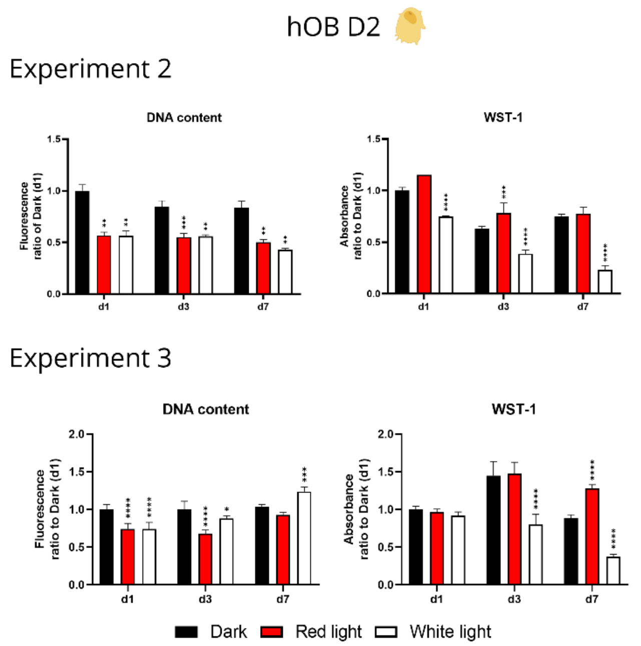

*Supplementary figure S5: Influence of red and white light illumination on hOB derived from donor 2 (D2) during cultivation over seven days – DNA content and metabolic activity, both normalised to the dark control on day 1, measured in two repeating experiments (experiments 2 and 3); n = 4, mean ± SD; p-values: *p < 0.05; **p < 0.01; ***p < 0.001; ****p < 0.0001.*

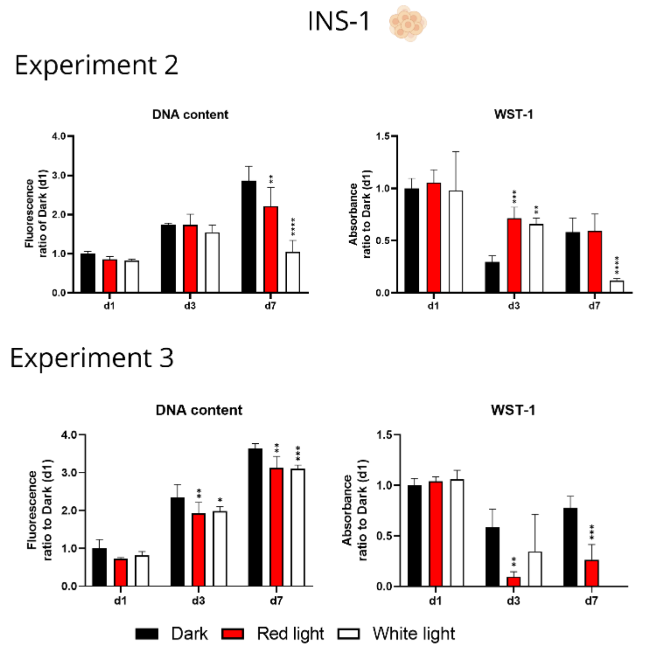

*Supplementary figure S6: Influence of red and white light illumination on INS-1 during cultivation over seven days – DNA content and metabolic activity, both normalised to the dark control on day 1, measured in two repeating experiments (experiments 2 and 3); n = 4, mean ± SD; p-values: *p < 0.05; **p < 0.01; ***p < 0.001; ****p < 0.0001.*

Experiment 2

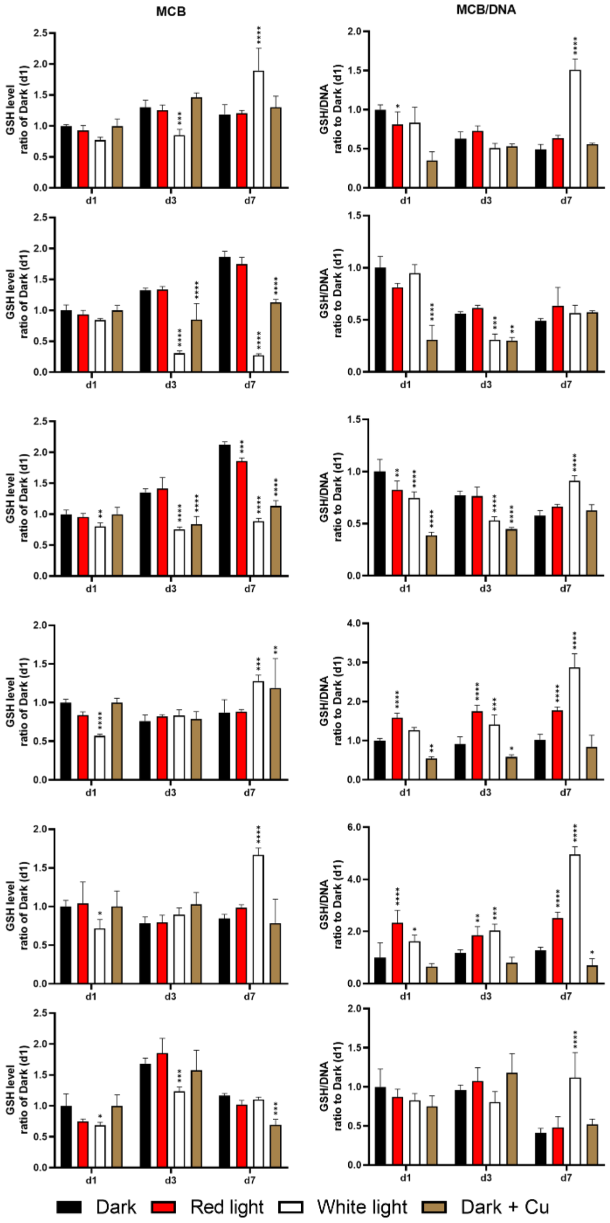

*Supplementary figure S7: GSH levels of all investigated cell types cultured under red and white light illumination, with a group cultivated in darkness as negative control and a positive control group with added copper ions in the standard cultivation medium – repeating experiment 2. Left side: GSH levels normalised to that measured for the dark control on day 1; right side: ratio of GSH level to DNA content of the same well, then normalised to the ratio determined for the dark control on day 1. n = 4, mean ± SD; p-values: *p < 0.05; **p < 0.01; ***p < 0.001; ****p < 0.0001.*

Experiment 3

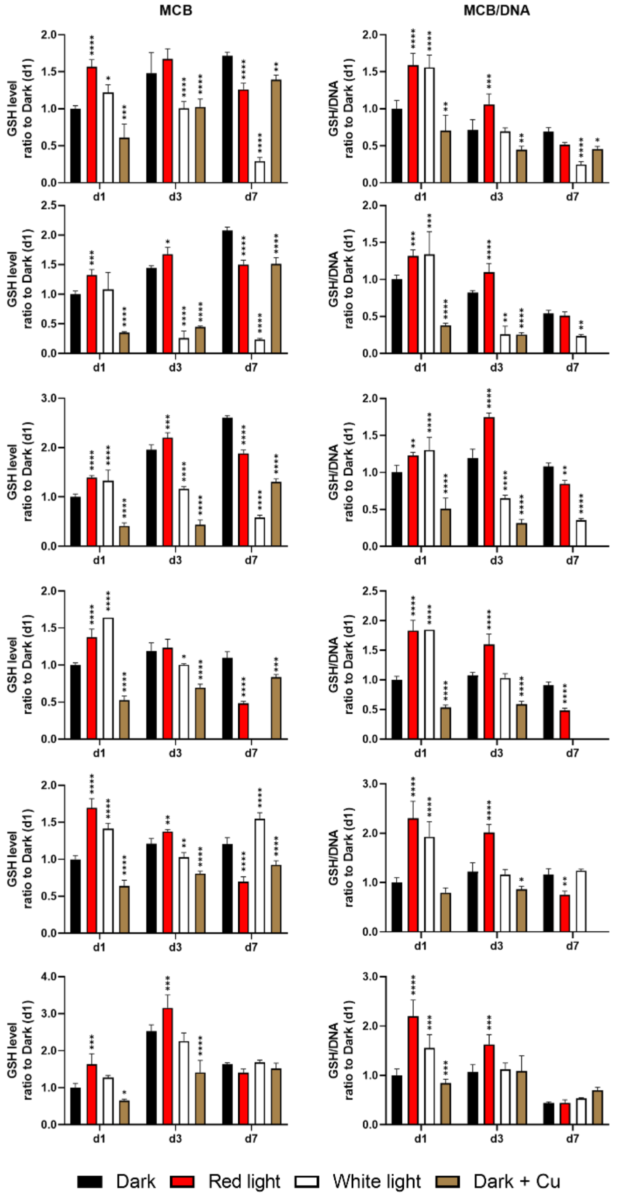

*Supplementary figure S8: GSH levels of all investigated cell types cultured under red and white light illumination, with a group cultivated in darkness as negative control and a positive control group with added copper ions in the standard cultivation medium – repeating experiment 3. Left side: GSH levels normalised to that measured for the dark control on day 1; right side: ratio of GSH level to DNA content of the same well, then normalised to the ratio determined for the dark control on day 1. n = 4, mean ± SD; p-values: *p < 0.05; **p < 0.01; ***p < 0.001; ****p < 0.0001.*

Light treated medium

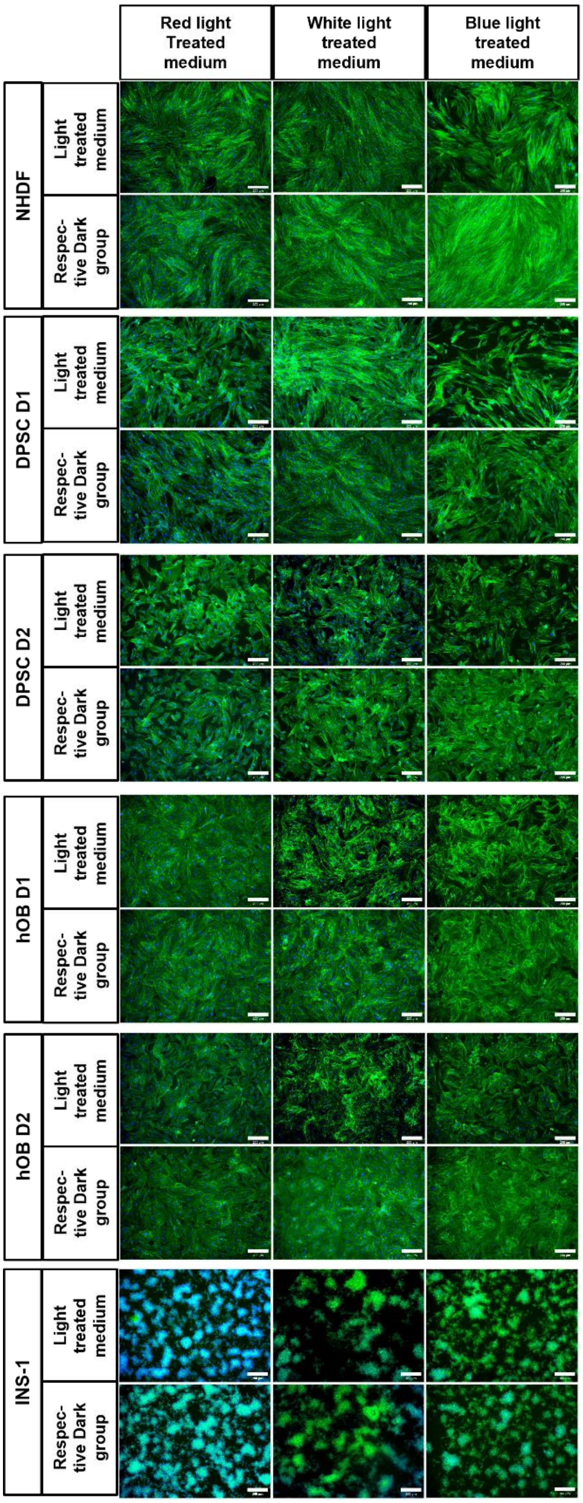

Supplementary figure S9: Influence of cell culture medium treated by red, white, or blue illumination on morphology and density of all investigated cell types – microscopy images of fluorescence staining (green – actin cytoskeletons, blue – cell nuclei) in comparison to the respective dark control group (medium which was not illuminated), day 3, scale bar: 200 µm.
